# Genomic Epidemiology of the Main SARS‐CoV‐2 Variants Circulating in Italy During the Omicron Era

**DOI:** 10.1002/jmv.70215

**Published:** 2025-02-12

**Authors:** Annalisa Bergna, Alessia Lai, Fabio Sagradi, Stefano Menzo, Nicasio Mancini, Bianca Bruzzone, Stefano Rusconi, Greta Marchegiani, Nicola Clementi, Daniela Francisci, Ilaria Vicenti, Silvia Ronchiadin, Harcel Djaya Mbissam, Carla della Ventura, Leonardo Lanfranchi, Sophie Testa, Sara Caucci, Carla Acciarri, Luca Carioti, Alessandro Occhionero, Federica Novazzi, Angelo Paolo Genoni, Francesca Drago Ferrante, Vanessa De Pace, Monica Ferraris, Matilde Ogliastro, Arianna Gabrieli, Massimo De Paschale, Giada Canavesi, Maria Concetta Bellocchi, Marco Iannetta, Loredana Sarmati, Francesca Ceccherini‐Silberstein, Agostino Riva, Spinello Antinori, Gianguglielmo Zehender, SARS‐CoV‐2 ITALIAN RESEARCH ENTERPRISE – collaborative Group, Claudia Balotta, Mario Corbellino, Massimo Galli, Valentina Ricucci, Federica Stefanelli, Nadia Randazzo, Giada Garzillo, Massimo Clementi, Maurizio Zazzi, Lia Fiaschi, Massimo Andreoni, Arianna Miola, Valeria Ricci, Laura Li Puma, Luigi Ruggerone

**Affiliations:** ^1^ Department of Biomedical and Clinical Sciences University of Milan Milan Italy; ^2^ Unit of Infectious Diseases Azienda Socio‐Sanitaria Territoriale Cremona Cremona Italy; ^3^ Department of Biomedical Sciences and Public Health Virology Unit, Polytechnic University of Marche Ancona Italy; ^4^ Department of Medicine and Technological Innovation University of Insubria Varese Italy; ^5^ Laboratory of Medical Microbiology and Virology Ospedale di Circolo e Fondazione Macchi Varese Italy; ^6^ Hygiene Unit IRCCS AOU San Martino‐IST Genoa Italy; ^7^ Ospedale Civile di Legnano ASST Ovest Milanese University of Milan Legnano Italy; ^8^ Department of Experimental Medicine University of Rome “Tor Vergata” Rome Italy; ^9^ Laboratory of Microbiology and Virology Università “Vita‐Salute” San Raffaele Milan Italy; ^10^ IRCCS San Raffaele Scientific Institute Milan Italy; ^11^ Department of Medicine and Surgery, Clinic of Infectious Diseases “Santa Maria della Misericordia” Hospital, University of Perugia Perugia Italy; ^12^ Department of Medical Biotechnologies University of Siena Siena Italy; ^13^ Intesa Sanpaolo Innovation Center‐ Artificial Intelligence Lab Turin Italy; ^14^ Clinic of Infectious Diseases – AOU delle Marche Ancona Italy; ^15^ Department of Health Sciences (DISSAL) University of Genoa Genoa Italy; ^16^ Unit of Microbiology Legnano Hospital, ASST Ovest Milanese Legnano Italy; ^17^ Department of System Medicine, Clinical Infectious Diseases Tor Vergata University Rome Italy

**Keywords:** COVID‐19 clinical aspects, genomic epidemiology, Omicron, phylogeny, SARS‐CoV‐2 variants

## Abstract

Since early 2022 the Omicron variant has rapidly spread worldwide, becoming the dominant variant to date. The study aimed to investigate the clinical and epidemiological characteristics of COVID‐19 patients and reconstruct the genomic epidemiology of main SARS‐CoV‐2 Omicron sublineages in Italy in 2022. A total of 8970 SARS‐CoV‐2 samples were studied, and phylogenetic analyses were focused on BA.1, BA.2, and BA.5 subvariants. More than half of subjects received three doses of vaccine and experienced a reinfection. A significant larger proportion of unvaccinated subjects presented reinfection compared with vaccinated. Clusters presented a tMRCA between September–November 2021 (BA.1), November 2021–January 2022 (BA.2), and October 2021–May 2022 (BA.5). *R*_e_ values showed the highest level between September–October, January–February 2022, and May 2022 for BA.1, BA.2 and BA.5, respectively. Limited number of studied variant sequences are included in clusters. The spread rate of the studied variant exceeded its evolutionary rate. No single sublineage had sufficient time to differentiate into large clusters, but only into small and fragmented groups sharing the same recent ancestor. These analyses dissect the epidemiological dynamics of Omicron sublineages in Italy over a period of great epidemiological changes in the COVID‐19 epidemic.

## 1 Introduction

COVID‐19 pandemic caused more than 665 million cases all over the world with more than 6,7 million of deaths by the end of 2022 (https://www.worldometers.info/coronavirus/).

The first genomes of SARS‐CoV‐2 were characterized and publicly shared already in January 2020 reaching by December 2022 the number of more than 15 millions genomes available for the scientific community (https://gisaid.org/). This large publicly available data, and the development of efficient methods for phylogenetic and phylodynamic analyses, allowed to track the evolution of the viral genome and identify emerging variants providing important public health tools for the surveillance of SARS‐CoV‐2 during the pandemic [1].

After the spillover event (or events) and the appearance of the first variant with the highest transmissibility (D614G) in 2020 [2], the virus circulated as a heterogeneous population of genomic sublineages all derived from the original lineages (called A and B, following the Pango classification).

The Variants of Concern (VOCs), carrying an unusual number of mutations, especially in the Spike protein and conferring to the mutant an increased transmissibility, were described for the first time in December 2020 (VOC Alpha, Beta, Gamma followed by Delta and then Omicron) and spread all over the world, with a mechanism of variant replacement [3].

At present, Omicron remains the dominant variant circulating globally, quite distantly related to previous VOCs [4] since it carries the highest number of mutations ever found in other VOCs. Moreover, it resulted associated with increased infectivity and enhanced immunoevasive properties [5]. Omicron was first identified in mid‐November 2021 in South Africa and was designated as VOC on November 26, 2021 (https://www.who.int/news/item/28-11-2021) [6]. However, retrospective analyses revealed that Omicron was present in Europe 10 days before its discovery in South Africa with no obvious transmission link between the two locations [7, 8].

Omicron comprises five distinct sublineages (BA.1‐5) that were discovered almost simultaneously, in November 2021, and each sublineage is different from the others as Alpha, Beta, Gamma, and Delta are far from each other [5]. It has been hypothesized that BA.4 and BA.5 may have diverged via a recombination event, with a suggested breakpoint between the E and M genes [9].

Epidemiological studies showed a global increase in the infection‐induced seroprevalence after the emergence of the Omicron variant in Europe [10, 11].

Thus, it is possible to identify a pre‐Omicron era, characterized by a low level of immunity in the population with the rise of variants with progressive increased transmissibility, and an Omicron era, characterized by the emergence of lineages with greater immunoevasive capacity selected by extensive natural and/or vaccine‐induced immunizaty [12, 13, 14].

It is debated whether Omicron has a lower virulence, however, the herd immunity due to previous infections and vaccinations observed during Omicron diffusion might strongly influence hospitalization and morbidity [15]. Convincingly, a recent WHO report showed evidence of reduced severity and lower mortality of the Omicron variant compared with the Delta variant after adjusting for the confounding effects of age, sex, ethnicity, prior infection, vaccination status, and comorbidities (https://www.who.int/publications/i/item/9789240051829). Both Pfizer and Moderna introduced an updated booster vaccine targeting Omicron sublineages to obtain higher effectiveness [16].

SARS‐CoV‐2 recombinants emerging during the different waves of COVID‐19 pandemic raised significant concerns, primarily due to their potential to accelerate immune evasion by means of antigenic shift. Among these recombinants, the first observed was named “Deltacron” [17], which originated in early 2022 from the recombination of Delta and Omicron BA.1 lineages, however, it exhibited limited spread. More recently, the newly identified XBB lineage, also called the Kraken variant [18], has gained considerable attention originating through the recombination of two highly diversified lineages, BJ.1 and BA.2.75.2, both arising from the Omicron BA.2 lineage. Remarkably, the XBB variants swiftly spread worldwide, infecting also subjects who had been vaccinated and/or with hybrid immunity. In September 2023, the new updated vaccine targeting Omicron XBB.1.5 became available [19]. Aims of this work were to study the clinical characteristics of COVID‐19 patients and to reconstruct the genomic epidemiology and phylodynamic of the main SARS‐CoV‐2 Omicron lineages circulating in Italy in 2022.

## 2 Materials and Methods

### 2.1 Sample Collection

Between January 1 and December 31, 2022, the Italian Centers participating in the SCIRE (SARS‐CoV‐2 Italian Research Enterprise) collaborative group characterized a total of 8970 SARS‐CoV‐2 positive samples obtained from either hospitalized or asymptomatic subjects tested in screening programs. The demographic characteristics of patients, as well as information about COVID‐19 vaccination status, hospitalization and SARS‐CoV‐2 genotype, were collected at each center for surveillance or for research purposes. This study was conducted in accordance with the principles of the 1964 Declaration of Helsinki and approved by the Sacco Hospital ethics committee (protocol n. 47866, September 9, 2020).

### 2.2 Virus Characterization

Variant assessment was performed by different methods: RT‐PCR variant specific screening assays (*n* = 4640, 51.7%), spike sequencing (*n* = 1164, 13%), and whole‐genome sequencing (WGS, *n* = 3166, 35.3%). Viral RNA extraction, RT‐PCR genotyping, amplification, and sequencing were obtained using different commercial kits or homemade procedures as previously described [20]. The SARS‐CoV‐2 lineage and clade were assigned to all Spike or Whole Genome (WG) sequences using the Pangolin COVID‐19 Lineage Assigner v. 4.3 (https://pangolin.cog-uk.io/) and Nextclade v. 2.14.1 (https://clades.nextstrain.org/). Mutations were identified using Nextclade.

### 2.3 Statistical Analysis

Statistical analyses were performed with the IBM SPSS Statistics version 29. Descriptive analyses of data are presented as a median and an interquartile range (IQR) when quantitative and as a proportion when qualitative. To compare normally distributed, nonnormally distributed continuous, and categorical variables, parametric tests (*t* test and ANOVA), nonparametric tests (Mann–Whitney and Kruskal–Wallis), and the Pearson 2 test (or Fisher exact test, when necessary) were used, respectively. A *p* value < 0.05 was considered statistically significant.

### 2.4 SARS‐CoV‐2 Data Sets

To study the major lineages of Omicron variant circulating during 2022, isolates of Omicron BA.1, BA.2, and BA.5 (BA.1, *n* = 268; BA.2, *n* = 677; BA.5, *n* = 713) were selected and aligned with other Italian sequences of the same lineage, available in GISAID (https://gisaid.org). Genomes were selected based on the following criteria: 10 whole genomes for each Italian region and sampling month, according with the circulation period of each subvariant, with a maximum of two sequences for region/week, excluding identical genomes and those with more than 5% of gaps. Three Italian data sets were set up: BA.1 (including a total of 880 isolates), BA.2 (*n* = 1627), and BA.5 (*n* = 1761) Omicron subvariants. The data set composition and regional distribution of Italian sequences are summarized in Tables S1 and S2. To place the Italian sequences in the international contest, an additional data set was set up for each variant, selecting five genomes for each European and non‐European countries and sampling month. Identical strains or those with more than 5% of gaps were excluded (Table S1).

Alignment of multiple sequences was obtained using MAFFT (https://mafft.cbrc.jp/alignment/server/) and the alignment was manually cropped using BioEdit v. 7.2.6.1 (https://bioedit.software.informer.com/). at the same length (29 774 bp).

The isolates included in the Omicron BA.1 data set dated between November 2021 and April 2022, the BA.2 subvariant isolates dated between January 2021 and December 2022, while the genomes included in the BA.5 subvariant data set had a date between April 2021 and December 2022.

### 2.5 Phylogenetic Analysis

The statistically significant clusters (including more than three sequences) were identified in the International ML trees by Cluster Picker v.1.2.3 using 70% bootstrap support and a mean genetic distance of 0.1% as thresholds. Epidemiological characteristics of the identified clusters were further investigated using Cluster Matcher v. 1.2.3 which allows the identification of clusters meeting given criteria. Clusters were classified as mixed (M), containing both Italian and non‐Italian isolates in different proportions, pure Italian (IT), including only Italian genomes, or European (EU), containing only European genomes.

The maximum likelihood trees of the three Italian data sets were estimated using IQ‐TREE v. 1.6.12 (http://www.iqtree.org/) [21]. The GTR + F + R3 (General time reversible + empirical base frequencies + three number of categories) model was used for BA.1 and BA.2 variants, while GTR + F + R6 models (General time reversible + empirical base frequencies + six number of categories) was used for BA.5. One thousand parametric bootstrap replicates were performed to support the nodes (≥ 60% bootstrap support).

For Italian data sets, the statistically significant clusters (including more than three sequences) were identified in the ML tree by Cluster Picker v.1.2.3 using 60% bootstrap support and a mean genetic distance of 0.1% as thresholds. Preliminary maximum likelihood tree was constructed including all the variants' significant clusters.

### 2.6 Phylodynamic Analysis

To characterize the epidemiological and evolutionary history of the different SARS‐CoV‐2 Omicron variants in Italy, only clusters including at least 10 sequences were considered for each Italian data set, by using the coalescent and the birth‐death models.

Bayesian analysis was performed by BEAST v. 1.10.4 (https://beast.community/) [22] with the same substitution model and molecular clock employed for the previously described analyses [23, 24]. Evolutionary rates were estimated using a Log Normal prior (mean, *M* = 8E‐4; variance, *S* = 1.25) in real space using a strict clock and Bayesian Skygrid model, a nonparametric coalescent model that estimates the effective population size over time.

MCMC (Markov chain Monte Carlo) analyses were run for 60 million generations and sampled every 3000. Convergence was assessed by estimating Effective Sampling Size (ESS) after applying a 10% burn‐in through Tracer v.1.7 software (http://tree.bio.ed.ac.uk/software/tracer/) [25], accepting ESS of at least 200. The uncertainty of estimates was indicated with 95% highest prior density (HPD) intervals.

The final tree was selected based on the maximum posterior probability (pp) value after performing a 10% burn‐in using Tree Annotator v.10.4 software (included in the BEAST package). Posterior probabilities greater than 0.7 were considered significant. Finally, all trees were visualized and edited in FigTree v. 1.4.4 (http://tree.bio.ed.ac.uk/software/figtree/).

The birth‐death skyline model implemented in Beast v. 2.7 was used to infer changes in the effective reproductive number (*R*_e_), and other epidemiological parameters such as the death/recovery rate (*δ*), the transmission rate (*λ*), the origin of the epidemic, and the sampling proportion (*ρ*) [26]. Given that the samples were collected during a short period of time, a “birth‐death skyline serial” model was used.

For the birth‐death analysis, one and two intervals and a lognormal before *R*_e_, with a mean (M) of 0.0 and a variance (S) of 1.8 were chosen, which allows the *R*_e_ values to change between less than 1 and more than 7.

A normal prior with *M* = 48.8 and *S* = 15 (IC95%: 24.0–73.4) was used for the rate of becoming uninfectious. These values are expressed as units per year and reflect the inverse of the time of infectiousness (5.3–19 days; mean, 7.5) according to the serial interval estimated by Li et al. [27]

Sampling probability (*ρ*) was estimated assuming a prior *β* (*α* = 1.0 and *β* = 1500), estimated based on available genomes in the analyses (normalizing to 1) and numbers of COVID‐19 active cases at pick of the studied period.

For all subvariants, origin of the epidemic was estimated using a lognormal prior with *M* = 0.1 and *S* = 0.3. The mean growth rate was calculated based on the birth and recovery rates (*r* = *λ* − *δ*), and the doubling time was estimated by the equation: doubling time = ln(2)/*r* [28].

## 3 Results

### 3.1 Population Characteristics

Analyzed samples were collected from Italian centers located in Liguria (*n* = 802), Lombardy (*n* = 5368), Umbria (*n* = 66), Marche (*n* = 2506), and Lazio (*n* = 228). Females accounted for 54% (*n* = 4788/8867) and the median age was 58 years (IQR: 37–76) without any significant differences between sexes. Significant differences were observed in the median age over different months (*p* < 0.001), with an increase in median age overtime (from 51 years in January to 73 years in December). Despite information of previous exposure to SARS‐CoV‐2 infection was available for a limited number of subjects (*n* = 478), 54.8% (*n* = 262) experienced a reinfection. Around one third of subjects had a known clinical status (*n* = 3102); 61.5% presented mild infections (*n* = 1908), followed by 31.7% of moderate/severe infections requiring hospitalization (*n* = 984). Among hospitalized patients, 6.3% (*n* = 62) required intensive care and 8 (0.8%) died. Hospitalized patients showed a higher median age compared with asymptomatic or mildly symptomatic subjects (*p* < 0.001, 73 vs. 52 and 49, respectively). Only a minority of the subjects were asymptomatics (210, 6.8%). These data are summarized in Table 1.

**Table 1:** Characteristics of studied subjects.

Characteristics	Overall
Study population, *n*(%)	8970
Sex, *n*(%)	
Males	4079 (46)
Females	4788 (54)
Median age (IQR)	58 (37–76)
Regions, *n*(%)	
Liguria	802 (9)
Lombardy	5368 (59.8)
Umbria	66 (0.7)
Marche	2506 (28)
Lazio	228 (2.5)
Non hospitalized	2118 (68.3)
Asymptomatic	210 (9.9)
Mild syndrome	1908 (90.1)
Hospitalized	984 (31.7)
Moderate/severe syndrome	914 (92.9)
Severe syndrome	62 (6.3)
Deaths	8 (0.8)
Reinfections	
yes	262 (54.8)
no	216 (45.2)
Vaccination status	
Vaccinated	2814 (67.3)
Unvaccinated	1365 (32.7)
Doses administered	
1	449 (16)
2	390 (13.9)
3	1030 (36.6)
4	61 (2.1)
Unknown	884 (31.4)
Type of vaccine	
Vaxzevria	22 (2.3)
Spikevax	220 (22.8)
Comirnaty	719 (74.7)
Jcovden	2 (0.9)

### 3.2 COVID‐19 Vaccinated versus Nonvaccinated

Among subjects with known COVID‐19 vaccination status (*n* = 4179), 67.3% (*n* = 2814) received at least one dose of vaccine. More than half of the studied subjects received three doses (53.4%, *n* = 1030/1930) of vaccine, only 3.1% received four doses (*n* = 61) and 78.8% received BNT162b2 vaccine (*n* = 719). The median time between the last dose of vaccine administered and infection was 6 months overall (IQR: 4–9). This value increased significantly (*p* < .00001) over the study period from 4 months (IQR: 2–8) in January to 12 months (IQR: 10–12) in December. Considering the number of received doses, significantly (*p* < .00001) longer times were observed among those who had received one (6 months, IQR: 1–11), two (7 months; IQR: 5–10) or three doses (7 months, IQR: 4–9) compared with those with four doses (4 months; IQR: 1–5).

No differences were observed in the proportion of vaccinated or nonvaccinated subjects in the study period, however, the median age of vaccinated individuals was lower compared with that of nonvaccinated ones (57.2 vs. 60, *p* < 0.001). A significant larger proportion of unvaccinated subjects presented reinfection compared with vaccinated (53%, 79/149 vs. 25.1%, 45/179; *p* < 0.001). Significant differences in the distribution of clinical status were present with the highest proportion of nonhospitalized subjects in vaccinated compared with unvaccinated (75.3%, 892/1,184 vs. 60.9%, 297/488; *p* < 0.0001). The proportion of deaths was significantly higher in unvaccinated than in vaccinated (0.6%, 3/488 vs. 0.4%, 5/1,184; *p* < 0.0001). No significant differences were observed in the gender distribution between vaccinated and unvaccinated subjects.

### 3.3 Lineages and Clades

**Figure 1:**
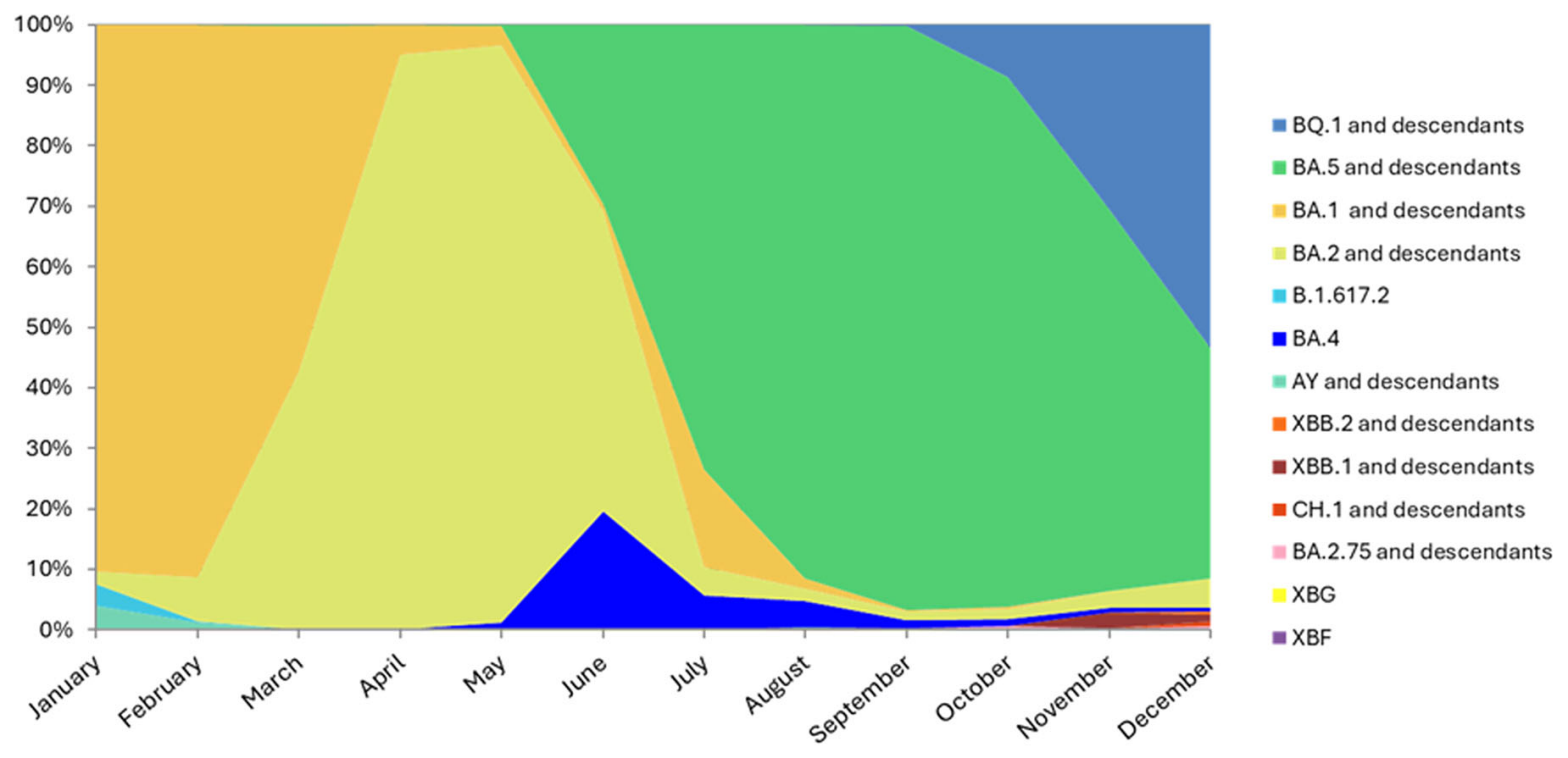
Dynamics of the SARS‐CoV‐2 epidemic in Italy, in term of lineages, during 2022.

The main observed variant in the studied cohort was Omicron (8740/8970, *n* = 97.4%) and its sublineages showing a prevalence of 44.6%, 26.8%, 1.8%, and 26.8% for BA.1 (*n* = 3898), BA.2 (*n* = 2338), BA.4 (*n* = 160), and BA.5 (*n* = 1654), respectively. The Delta variant was observed until August, when the last case was observed, and globally accounted for 1.8% (*n* = 161). Recombinants represented 0.7% (*n* = 61) of total sequences and included XC (*n* = 39), XAZ (*n* = 1), XBB (*n* = 16), XBG (*n* = 1), XBF (*n* = 1), XQ (*n* = 1), and XT (*n* = 2). First cases of XBB recombinants were observed in November (*n* = 10, 2.6%). BA.1 remained the dominant until March (90.3%, *n* = 2241; 91.2%, *n* = 869; and 57.1%, *n* = 628 in January, February, and March, respectively) and completely disappeared since November. From April (95.2%, 867/911) to June (48.6%, 232/477), BA.2 became prevalent and was then replaced by BA.5. BA.5 reached the highest prevalence in September (96.7%, 384/397) and then decreased to 44.1% (162/367) in December when BQ.1 and descendants prevailed (52.6%, *n* = 193). First case of BQ.1 was observed in September (Table S3). Accordingly, the main clades included 21 K (39.7%, *n* = 3469), 21 L (25.4%, *n* = 2279), and 22B (22.6%, *n* = 2030) (Figure 1). By considering clinical status between subjects infected with Delta versus Omicron variant, a significantly higher proportion of nonhospitalized subjects was observed in subjects infected by Omicron (68.4%, 2091/3056 vs. 58.7%, 27/46; *p* = 0.017) and a significant higher proportion of deaths was found in Delta patients (2.2%, 1/46 vs. 0.2%, 7/3,056; *p* = 0.017). Of note, considering vaccination status, significant higher proportions of hospitalization and deaths were present in vaccinated patients carrying Delta variant compared with vaccinated or unvaccinated subjects with Omicron (72%, 18/25 vs. 23.2%, 269/1159 and 38.8%, 185/477 for hospitalization and 4%, 1/25 versus 0.3%, 4/1159 and 0.6%, 3/477 for deaths, *p* = 0.02). Considering Delta versus Omicron sublineages, the highest proportions of hospitalization were observed in Delta and BA.5 compared with BA.1 and BA.2 (53.1%, 17/32 and 51.5%, 135/262 vs. 11.8%, 52/442 and 28.8%, 242/839; *p* < 0.0001), while the proportion of deaths was significantly higher in subjects affected by Delta compared with those by Omicron sublineages (3.1%, 1/32 vs. 1.4%, 6/442, 0 and 0.4%, 1/262; *p* < 0.0001). In accordance with the circulation period of the different variants, the median time from vaccination to infection was significantly (*p* < .00001) longer for the BQ.1 variant (11 months; IQR: 9–12) and recombinant lineages (12, IQR: 11–12) than for the Delta (3; IQR: 2–6) and BA.1 variants (4; IQR: 2–7).

### 3.4 Mutation Analyses of the Italian Sequences

Table S4 shows the sublineage composition of Italian Omicron BA.1 data set.

The comparison between genomes from Italy and the reference sequence showed 49 aminoacidic substitutions and 7 deletions present in at least 10% of isolates. More than 30 mutations were present in the spike protein. Over 90% of the sequences had characteristics mutations of this variant and its descendants (Table S5). The V1187I mutation in ORF1a, characteristic of sublineages BA.1.17 and BA.1.17.2, was globally found in 32.4% (*n* = 285) of genomes but was present in 90.5% (67/74) and 97.6% (204/209) of BA.1.17 and BA.1.17.2, respectively. The R346K mutation in the spike protein, found in 39.2% (*n* = 345) of sequences, was present in 94.8% (*n* = 275) of BA.1.1 genomes (*n* = 290) and in almost all (88.4%, *n* = 61) BA.1.1.1 (*n* = 69) and descendants isolates. The G446S mutation in the Spike protein, typical of the BA.1.1 and descending sublineages, was found in a total of 665 isolates (75.6%), of which 73.3% (*n* = 545) of BA.1.1 and descendants (*n* = 746). The A701V mutation in the Spike protein, present in 20.5% (*n* = 180) of genomes, was found in 85.6% (179/209) of the sequences belonging to this sublineage BA.1.17.2. In addition, the totality of the sequences of sublineages BA.1.15 and descendants (17/17), had the additional mutation D343G in the protein N, distinctive of this sublineage.

The sublineage composition of the sequences included in the Omicron BA.2 data set was shown in Table S6.

In the BA.2 and descendants data set, only mutations/deletions typical of this lineage were found, as shown in Supporting Information (Table S7). In the ORF3a region, the H78Y mutation, present in a total of 16.5% (*n* = 268) of the isolates, was prevalent in the BA.2.9 sublineage isolates (88.5%, 234/265). In the ORF1a region C655R, A2909V and Q3966H mutations (observed in less than 5% in data set global) were identified in almost 100% of the isolates of sublineages BA.2.52.2 (33/33), BA.2.3 (44/44), and BA.2.22 (16/18), where these substitutions are characteristic. Similarly, S959P mutation in ORF1b, was present in 95.2% (20/21) of the BA.2.10 isolates. The L140F mutation in ORF3a, was found in almost all BA.2.3 and descendant sequences (97.7%, 42/43). In protein S, two mutations characteristics of the sublineage BA.2.12.1, L452Q and S704L, were identified in 94.5% (63/66) and 98.5% (65/66) of its isolates, respectively.

Table S8 shows the sublineage composition of Omicron BA.5 data set.

A total of 50 mutations and 5 deletions were found in at least 10% of the sequences analyzed (Table S9). These substitutions have been identified in more than 80% of the isolates, except for the T1050N mutation, in the ORF1b region, with a global prevalence of 20.8% (*n* = 367) but which was present in almost all isolates BA.5.2, BA.5.2.2 and descendant sublineages, and mutation D16G in the ORF9b region; this substitution, present in almost 50% (*n* = 844) of the isolates, was found in all BA.5.2 isolates (*n* = 350) and in 97.6% (322/330) of BA.5.2.1 sequences. In protein S, in addition to mutations typical of this variant, 98.1% (*n* = 1,721) of the sequences bore the mutation G142Y. Substitutions with a global frequency of less than 10% but characteristics of different sublineages have been identified in the ORF1a, ORF1b, S and N regions. In the ORF1a region, mutations S302F, Q556K, K3839R and T4161I were observed in all BA.5.1.23 (*n* = 27), BA.5.3.1 (*n* = 16), BA.5.1.10 (*n* = 101), and BA.5.1.8 (*n* = 37) sequences, respectively.

### 3.5 Phylogenetic Analysis of International Data Sets

Maximum Likelihood analysis of the international data sets showed that the majority of whole genomes of BA.1, BA.2, and BA.5 (ranging from 72.5% to 87.6%) were scattered throughout the trees, while 12.4% (228/1837) of BA.1, 19.9% (649/3246) of BA.2, and 27.5% (885/3219) of BA.5 genomes formed significant clusters, from 3 to 17, 30 and 60 sequences (for BA.1, BA.2 and BA.5, respectively) and mainly localized at the external nodes of the trees. In detail, 46.2% (24/52) of BA.1 clusters included sequences exclusively from Italy, as well as 37.9% (53/140) of BA.2 clusters and 36.5% (69/189) of BA.5 clusters, while mixed clusters were 9.6% (5/52), 27.9% (39/140), and 49.7% (94/189), respectively.

### 3.6 Phylogenetic Analysis and Dating of Italian Clusters

The phylogenetic analysis conducted on the Italian sequences of the subvariant Omicron BA.1 showed the presence of 30 clusters, characterized by more than three sequences (min 4–max 21), which included 24.3% (*n* = 214) of total analyzed sequences (*n* = 880); four (8.7%) clusters included more than 10 genomes. There was no change in the pattern of clustering based on the sampling area (northern, southern, central Italy, and islands).

Analysis of the Omicron BA.2 subvariant showed the presence of 60 clusters (min–max: 4–30 sequences), which included 22.6% of the isolates analyzed (368/1627); 7 (6.3%) clusters included more than 10 genomes. No different clustering pattern was found based on the sampling area.

The 26.5% (*n* = 467) of isolates included in the BA.5 data set (*n* = 1761) grouped into 68 clusters, of which 10 (7.4%) clusters included a number greater/equal to 10 genomes. Sequences from the islands clustered more frequently than those from northern, central, and southern Italy (52.3% vs. 37.6%, 35.6%, 37.2%; *p* < 0.05).

Maximum Likelihood analysis conducted on isolates included in all BA.1, BA.2, and BA.5 clusters showed that these lineages formed three highly significant monophyletic groups (Table S10 and Figure S1).

Preliminary analysis by root‐to‐tip regression revealed a linear relationship between genetic diversity and time (correlation coefficient = 0.81 and *R* ^2^ = 0.66) (Figure S2).

Given the limited number of BA.1 clusters containing more than 10 isolates, the Bayesian phylogenetic analysis was conducted on a data set that included all sequences forming clusters with at least four sequences (*n* = 214).

Bayesian analysis estimated a mean substitution rate of 4.84 × 10^‐4^ s/s/y (95%HPD: 3.76–5.98 × 10^‐4^ s/s/y) and showed that all sequences grouped within 12 statistically supported clusters (pp > 0.9) in the tree (Figure S3).

The tMRCA of each cluster was dated between September and November 2021 (95% HPD: June–December 2021) (Table 2). These clusters contained an average of 17.8 genomes (minimum of 4 and maximum of 71) with a persistence between 2 and 7 months (Table 2). Earlier clusters (dated September 2021) showed the larger size (20.5 vs. 6.5 isolates) and the longer persistence (7 vs. 4.5 months) than later clusters (dated October/November).

**Table 2:** tMRCA estimation of the main clusters of BA.1 data set with the relative 95% HPD and duration in the time.

	n. of sequences	*pp*	Data	95%HPD *Lower*	95%HPD *Upper*	More recent samples	Months
#1	71	0.98	05/09/2021	21/06/2021	23/10/2021	05/04/2022	7
#11	20	0.99	06/09/2021	02/07/2021	29/10/2021	04/04/2022	7
#10	16	0.97	14/09/2021	13/07/2021	02/11/2021	31/01/2022	4
#12	16	0.99	16/09/2021	21/07/2021	04/11/2021	08/02/2022	5
#8	21	0.99	20/09/2021	16/07/2021	05/11/2021	02/04/2022	7
#7	21	0.92	25/09/2021	21/07/2021	09/11/2021	03/04/2022	7
#9	9	0.99	02/10/2021	04/08/2021	14/11/2021	07/04/2022	6
#2	19	1	11/10/2021	30/08/2021	13/11/2021	28/03/2022	5
#6	7	1	23/10/2021	14/09/2021	26/11/2021	12/03/2022	5
#3	6	1	31/10/2021	28/09/2021	29/11/2021	28/12/2021	2
#5	4	1	03/11/2021	01/10/2021	03/12/2021	31/01/2022	2
#4	4	1	06/11/2021	01/10/2021	09/12/2021	07/03/2022	4

Abbreviations: HPD, highest posterior density; pp, posterior probability.

Bayesian phylogenetic analysis was conducted on BA.2 data set that included all sequences included in the seven largest Italian clusters containing more than 10 sequences for a total of 111 genomes.

The Bayesian analysis estimated a mean evolutionary rate of 3.99 × 10^‐4^s/s/y (95%HPD: 2.70–5.33 × 10^‐4^).

Clusters contained an average of 15.9 genomes (min–max: 10–30), dated between November 2021 and January 2022 (95%HPD: August 2021–February 2022) and a persistence between 4 and 8 months (Figure S4; Table 3) without any relationship between the clusters size and persistence.

**Table 3:** tMRCA estimation of the main clusters of BA.2 data set with the relative confidence intervals and duration in the time.

	n. of sequences	*pp*	data	95%HPD *Lower*	95%HPD *Upper*	More recent samples	Months
#249	11	0.99	26/11/2021	28/08/2021	30/01/2022	07/06/2022	7
#114	12	0.99	11/12/2021	16/10/2021	22/01/2022	07/06/2022	5
#40	16	0.99	27/12/2021	13/11/2021	03/02/2022	08/09/2022	8
#52	30	1	01/01/2022	17/11/2021	08/02/2022	07/06/2022	5
#133	10	1	03/01/2022	25/11/2021	05/02/2022	04/05/2022	4
#117	17	1	03/01/2022	28/11/2021	05/02/2022	07/06/2022	5
#57	15	1	13/01/2022	29/11/2021	23/02/2022	04/07/2022	6

Abbreviations: HPD, highest posterior density; pp, posterior probability.

Bayesian analysis of the Omicron BA.5 variant was conducted on the 164 sequences included in the 10 clusters containing more than 10 sequences.

The estimated evolutionary rate showed an average of 4.56 × 10^‐4^ s/s/y (95%HPD: 3.72 × 10^‐4^–5.44 × 10^‐4^). Clusters' tMRCAs dated from October 2021 to May 2022 (95%HPD: July 2021–July 2022) (Figure S5), but most of them dated in March and April 2022. Clusters contained an average of 16.4 genomes (min–max: 10–39) and showed a persistence of a mean 8.3 months (range: 5–11 months) (Table 4). No relationship was observed between the clusters size and persistence.

**Table 4:** tMRCA estimation of the main clusters of B.5 data set with the relative confidence intervals and duration in the time.

	n. of sequences	*pp*	data	95%HPD *Lower*	95%HPD *Upper*	More recent samples	Months
#208	11	0.99	24/10/2022	25/07/2021	16/01/2022	25/07/2022	9
#141	18	0.99	08/01/2022	02/11/2021	08/01/2022	08/11/2022	11
#42	20	1	13/02/2022	21/12/2021	04/04/2022	10/12/2022	10
#113	21	1	21/03/2022	01/02/2022	04/05/2022	07/11/2022	8
#92	39	1	26/03/2022	02/02/2022	11/05/2022	09/12/2022	9
#105	12	1	31/03/2022	05/02/2022	17/05/2022	17/12/2022	9
#133	10	0.99	05/04/2022	16/02/2022	18/05/2022	21/11/2022	7
#192	10	1	17/04/2022	13/03/2022	20/05/2022	27/09/2022	5
#62	10	1	20/04/2022	11/03/2022	28/05/2022	13/12/2022	8
#213	13	1	24/05/2022	15/04/2022	14/07/2022	15/12/2022	7

Abbreviations: HPD, highest posterior density; pp, posterior probability.

### 3.7 Bayesian Phylodynamic Analysis

**Figure 2:**
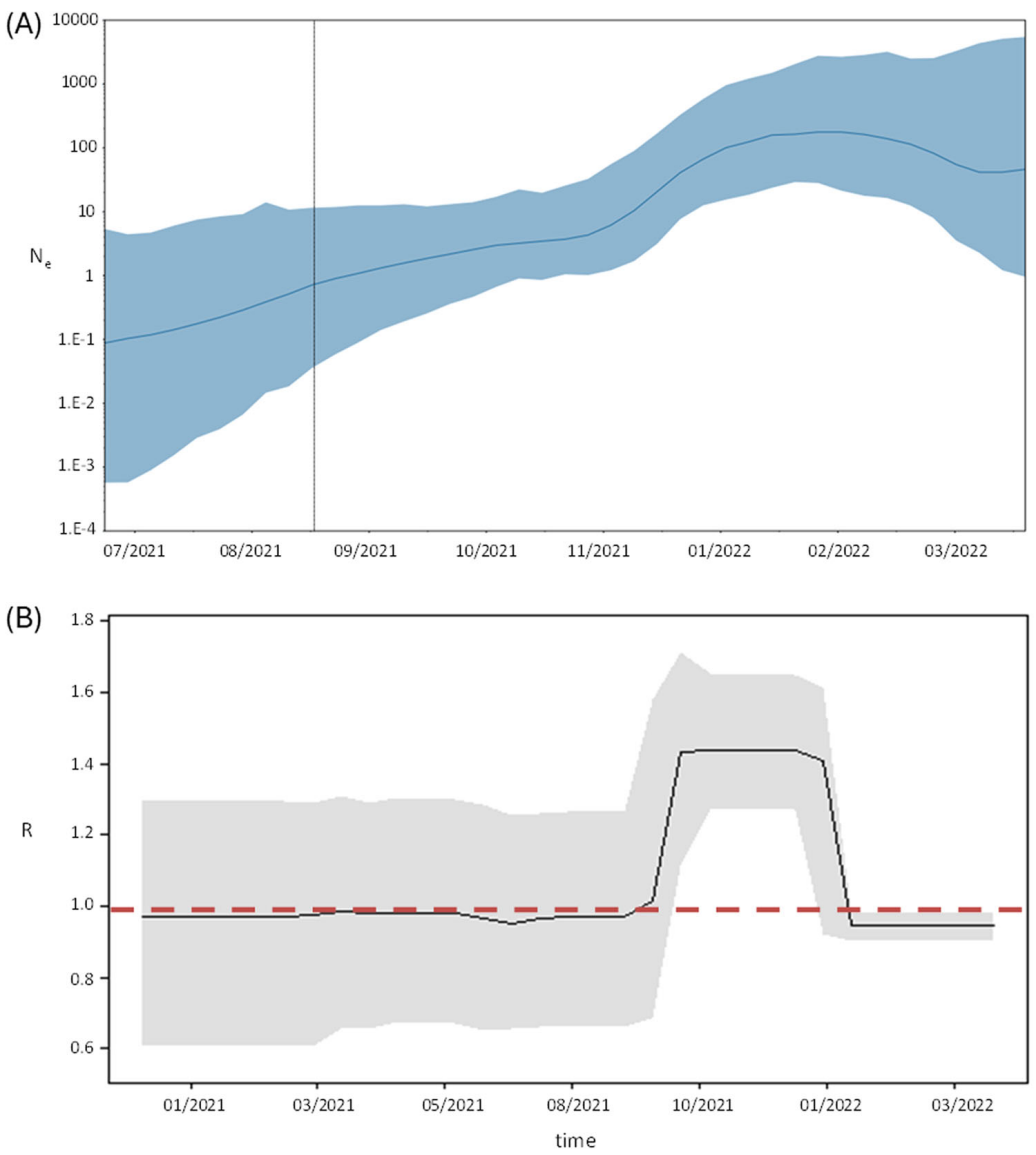
(A) Bayesian Skygrid plot of BA.1 variant. The *y*‐axis indicates the effective population (Ne), the *x*‐axis shows the time expressed in dates. The thick line in the graph indicates the median of the value of the estimate, while the blue area indicates 95% HPD. (B) Birth‐death skyline plot of BA.1 variant, in relation to time (*x*‐axis) and the effective reproduction rate (*R*_e_) (*y*‐axis).

The Bayesian phylodynamic analysis of the BA.1 Italian clusters, showed that the number of infections progressively grew since the origin of the epidemic (September 2021); a spike growth started in November 2021 reaching the plateau in January 2022 lasting until March 2022, when the effective number of infections started to decrease (Figure 2A).

In agreement with this dynamic, the estimate of *R*_e_ was close to the threshold 1 until December when the effective reproduction number reached 1.45, followed by a decline in January 2022 (when the number of infections reached the plateau) to the initial values (Figure 2B).

**Figure 3:**
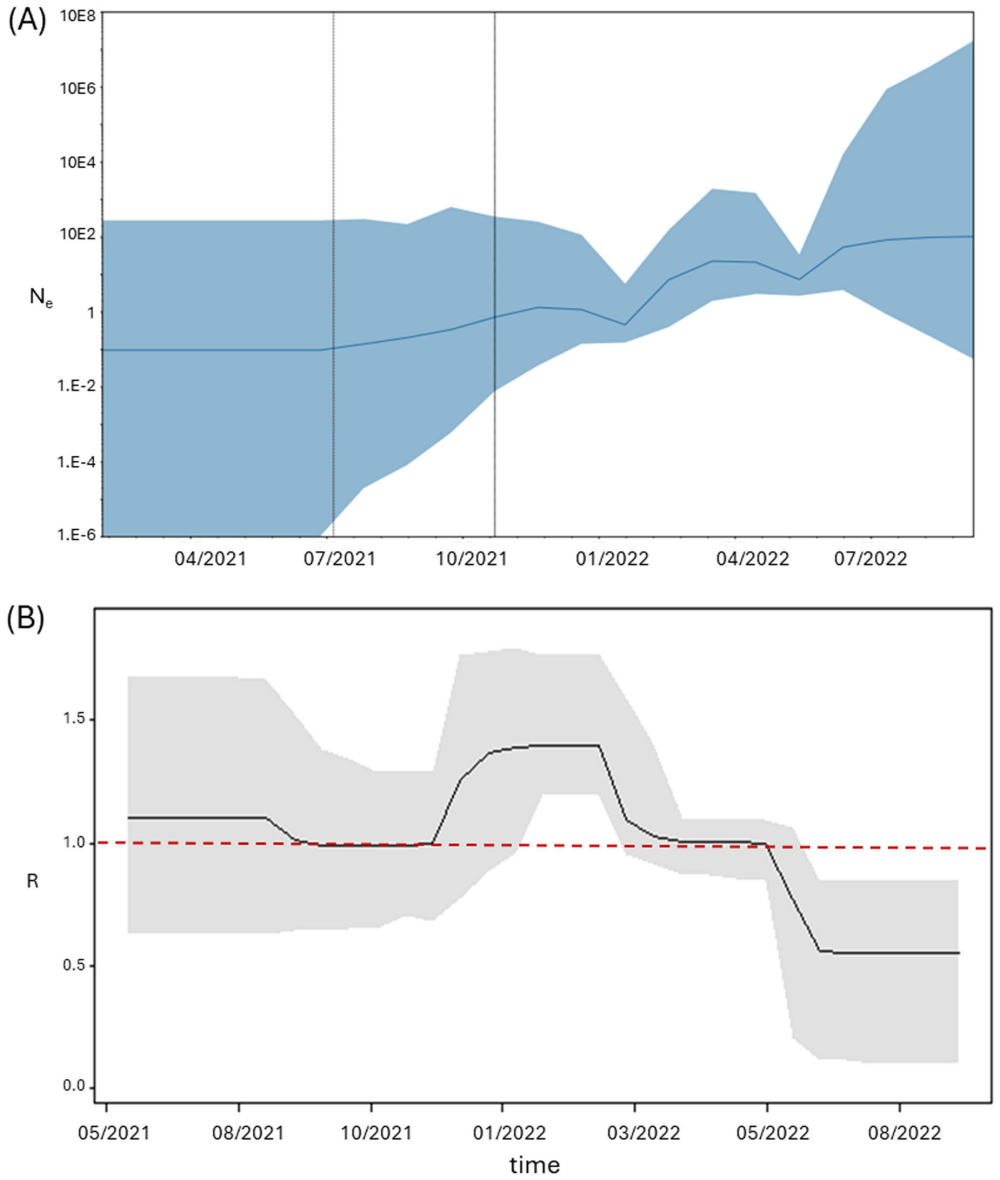
(A) Bayesian Skygrid plot of BA.2 variant. The *y*‐axis indicates the effective population (Ne), the *x*‐axis shows the time expressed in dates. The thick line in the graph indicates the median of the value of the estimate, while the blue area indicates 95% HPD. (B) Birth‐death skyline plot of BA.2 variant, in relation to time (*x*‐axis) and the effective reproduction rate (*R*_e_) (*y*‐axis).

In the case of BA.2, an exponential increase of the effective number of infections was observed only in January/February 2022 and the plateau was reached between March and April 2022, when an initial decline in the number of infections was observed, followed by a rebound during summer (Figure 3A). Similarly, the estimate of the *R*_e_ has shown values around 1 since the beginning of the epidemic, but the peak (1.42) was reached between January and February 2022, returning to values around the unity in February–March showing a more pronounced reduction between May and July 2022 (Figure 3B).

**Figure 4:**
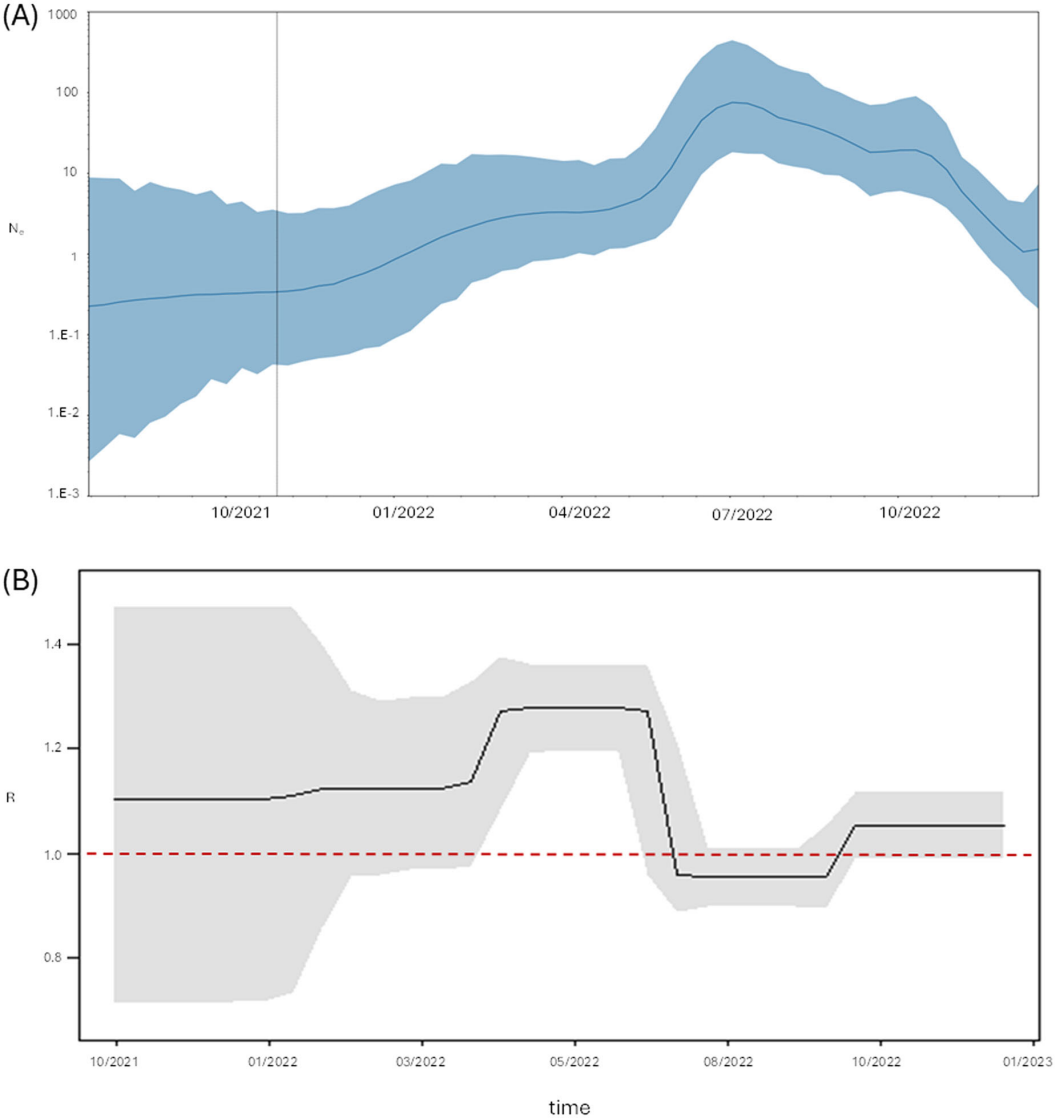
(A) Bayesian Skygrid plot of BA.5 variant. The *y*‐axis indicates the effective population (Ne), the *x*‐axis shows the time expressed in dates. The thick line in the graph indicates the median of the value of the estimate, while the blue area indicates 95% HPD. (B) Birth‐death skyline plot of BA.5 variant, in relation to time (*x*‐axis) and the effective reproduction rate (*R*_e_) (*y*‐axis).

The curve showing the effective number of BA.5 infections exhibits two growth phases, with the initial phase in January 2022, being flatter, followed by a subsequent steeper increase starting from May 2022 and reaching a peak of cases around July. The decrease began in the second half of the same month of July or August, with a more pronounced decline starting from October 2022 (Figure 4A).

Similarly, the estimate of the *R*_e_ showed values above 1 from October 2021, although the highest values were observed from May 2022 (1.28) to July, when the estimates of the effective reproduction number dropped around 1, where they remained until the end of the study (Figure 4B).

## 4 Discussion

Since early 2022 the Omicron variant has rapidly spread worldwide, becoming up to now the dominant variant with its derived sublineages [29, 30]. The higher transmissibility, the lower neutralizing efficacy of antibodies stimulated by previous infections or vaccination, the less severe clinical spectrum, the shorter incubation period, and the higher replicative efficiency, made this variant very different from those that circulated previously. In addition, the widespread diffusion of vaccination leading to over 85% of the Italian population vaccinated with a complete cycle (https://www.lombardianotizie.online/vaccinati-over-80/), and the progressive easing of containment measures up to their total elimination, radically changed the epidemiology of the infection leading to a distinction between a past “pre‐Omicron” and a current Omicron era [13, 31].

According to the study period, the Omicron variant was highly prevalent in this work, representing more than 97% of viral detections while the Delta variant was observed in less than 2% of the subjects up to August, when it disappeared similarly to what observed in the rest of the world. A similar replacement trend was also observed in the succession of Omicron sublineages overtime. Lineage BA.1 was prevalent until March 2022, when it was replaced by BA.2 which remained the dominant lineage until June, when BA.5 became prevalent until November. At the end of the study the lineage BQ.1, derived from BA.5, became the most frequent.

While the BA.1 variant became extinct a few months after its spread, BA.2 and BA.5 variants continued (February 2024) to circulate with different derived sublineages and recombinant forms favored by the increased transmissibility [32]. The circulation of recombinants started in 2022 with the co‐circulation of Delta and Omicron VOCs, however, their large spread matched with the identification of XBB recombinants derived from recombination between two lineages of BA.2, which were first identified only at the end of study period (November 2022).

Due to the predominant enrollment of patients from clinical centers and related microbiology laboratories, only a small number of asymptomatic subjects were included. Vaccination coverage with at least one dose of anti‐SARS‐CoV‐2 vaccine was significantly lower in enrolled subjects compared with the national coverage.

Despite this bias, the unvaccinated subjects more frequently required hospitalization compared with vaccinated (24.7% among vaccinated vs. 39.1% among unvaccinated), unvaccinated also experienced a doubled frequency of reinfections and a higher frequency of deaths, confirming previous data on the efficacy of COVID‐19 vaccines. Moreover, these data confirm that Omicron is less virulent than the Delta variant but also indicated, differently from previous reports [33, 34], a lower lethality both in vaccinated and unvaccinated subjects. Despite the small number of individuals infected by Delta variant in the study, a relevant difference in the proportion of deaths was also observed comparing with different Omicron sublineages. A relevant factor possibly influencing these observations could be the changed criteria adopted for the admission of COVID‐19 patients during the study period by the clinical centers. As vaccination became more spread in the population, the COVID‐19 pressure on the healthcare system was alleviated allowing the structures to admit also less severe infections. Mitigation of prophylactic measures also favored the nosocomial circulation of the virus in patients and health workers. These phenomena are clearly testified by the decreasing age of the enrolled subjects throughout the course of the study. This kind of bias probably also applies to all other studies describing clinical differences between SARS‐CoV‐2 variants, given the impossibility to set‐up rigorous prospective studies.

Moreover, although this work is the result of the collaboration of many clinical/diagnostic centers located throughout the country, some regions are less represented leading to a potential sampling bias [35, 36]. However, the study included more than 8000 samples collected during the year 2022 in five Italian regions accounting for more than 33% of the Italian population (19 million out of a total population of 58 million individuals in 2024); two regions comprising at least 72.5% of the population living in North‐Western (around 72 million out of 115 million inhabitants) and three in the central part of the Country, accounting for 68% (8 million out of 69 million, data from ISTAT: http://dati.istat.it/Index.aspx?DataSetCode=DCIS_POPRES1).

Compared with previous variants, the Omicron variant displayed a high number of mutations, with an average of 50 substitutions throughout the genome, mainly in the spike protein. These characteristics are responsible for a higher binding affinity to the ACE2 receptor and greater immune escape from neutralizing and therapeutic monoclonal antibodies [12, 14, 32]. This variant has rapidly further evolved giving rise to large array of multiple lineages and sublineages [37] with specific mutational profiles.

By analyzing the mutational profile of these sublineages, we found a high number of mutations, mainly located in the S gene, all identical to those previously described.

The phylogenetic analysis of the international data sets showed only the presence of small clusters at the external nodes of the tree, including few isolates probably closely epidemiologically related, thus preventing the observation of larger significant transmission clusters, as reported in other study [38]. This was due to the scanty, relatively rare, and dispersed sampling, along with the strategies adopted to reduce the magnitude of genomes introduced in the analysis.

It is also likely that, as the spread rate of the variant exceeded its evolutionary rate (Omicron became prevalent worldwide within a couple of months) thanks to its escape to natural and vaccine‐induced immunity and to the lowering of restrictions, single sublineage did not have sufficient evolutionary pressure for being selected to form large clusters, but only small and fragmented groups sharing the same recent ancestor [39, 40].

Analyzing the subvariants within the international context, there was a tendency for Italian isolates to group in the tree in the same regions, even without forming distinct and significant clades. For this reason, the phylogenetic analysis was conducted considering only the national context in which we found a partial formation of clusters on a local basis, mainly in the case of the BA.1 and BA.2 lineages.

Indeed, thanks to the neutral evolution phenomena, such as the “founding effect,” it was possible to identify significant clusters only at local (national) level, while in the international context, due to the high degree of evolutionary correlation among strains of a single variant (BA.1, BA.2, and BA.5), it is difficult to identify more closely related groups of sequences that share a recent common ancestor [39, 40].

The tMRCA for BA.5 suggests that this lineage would have been circulating throughout the period dominated by BA.1 and then BA.2 without any transmission advantage. According to literature data, maximum likelihood estimations suggest that BA.5 could have descended from BA.2 [9].

For these subvariants, higher *R*_e_ values were observed than those estimated for previous variants [23], confirming Omicron's enhanced transmissibility. Although the literature data indicate a higher transmissibility of BA.2 than BA.1 [41, 42], the values we estimated were comparable. These estimates are in line with the official ones (https://covid19.infn.it/), except for the peak in January 2022, corresponding to the simultaneous circulation of the Delta and Omicron variants. Although the mean *R*_e_ value estimated at peak for BA.5 was lower than those estimated for BA.1 and BA.2, there was a persistence of values above 1 over time since the beginning of the epidemic, which would account for the peak of infected individuals observed in the 2022 wave (https://covid19.infn.it/).

Analyses show a good temporal correspondence between the trend in the number of infections estimated from the Skyline graph and the estimated *R*_e_ by birth‐death Skyline.

In conclusion, these data allow an accurate description of the epidemiological dynamics of Omicron sublineages in Italy over a period of great epidemiological changes in the COVID‐19 epidemic.

## Collaborative Group

SCIRE collaborative Group: Claudia Balotta, Mario Corbellino, Massimo Galli, Valentina Ricucci, Federica Stefanelli, Nadia Randazzo, Giada Garzillo, Massimo Clementi, Maurizio Zazzi, Lia Fiaschi, Massimo Andreoni, Arianna Miola, Valeria Ricci, Laura Li Puma, Luigi Ruggerone.

## Author Contributions

Annalisa Bergna, Alessia Lai, Gianguglielmo Zehender conceived the project; Fabio Sagradi, Stefano Menzo, Nicasio Mancini, Bianca Bruzzone, Stefano Rusconi, Greta Marchegiani, Nicola Clementi, Daniela Francisci, Ilaria Vicenti, Silvia Ronchiadin, Carla della Ventura, Leonardo Lanfranchi, Sophie Testa, Sara Caucci, Carla Acciarri, Luca Carioti, Alessandro Occhionero, Federica Novazzi, Angelo Paolo Genoni, Francesca Drago Ferrante, Vanessa De Pace, Monica Ferraris, Matilde Ogliastro, Arianna Gabrieli, Massimo De Paschale, Giada Canavesi, Maria Concetta Bellocchi, Marco Iannetta, Loredana Sarmati, Francesca Ceccherini‐Silberstein, Agostino Riva, Spinello Antinori collected the samples and information; Alessia Lai, Harsel Djaya Mbissam performed statistical data analyses; Annalisa Bergna, Alessia Lai, Gianguglielmo Zehender performed phylogenetic analyses; Annalisa Bergna, Alessia Lai, Gianguglielmo Zehender interpreted the results; Fabio Sagradi, Bianca Bruzzone, Greta Marchegiani, Nicola Clementi, Ilaria Vicenti, Carla della Ventura, Leonardo Lanfranchi, Sara Caucci, Carla Acciarri, Luca Carioti, Federica Novazzi, Angelo Paolo Genoni, Francesca Drago Ferrante, Vanessa De Pace, Monica Ferraris, Matilde Ogliastro, Massimo De Paschale, Giada Canavesi, Maria Concetta Bellocchi contributed to the sequencing; Annalisa Bergna, Alessia Lai, Stefano Menzo, Gianguglielmo Zehender wrote the first draft of the manuscript. All authors read, revised and approved the final version of the manuscript.

## Conflicts of Interest

The authors declare no conflicts of interest.

## Supporting information

Supporting information.

## Data Availability

All analytical data are available within the article, Figures, and Supplementary Data. Whole‐genome sequences were submitted to GISAID.

## References

[1] S. W. Attwood , S. C. Hill , D. M. Aanensen , T. R. Connor , and O. G. Pybus , “Phylogenetic and Phylodynamic Approaches to Understanding and Combating the Early SARS‐CoV‐2 Pandemic,” Nature Reviews Genetics 23, no. 9 (2022): 547–562. 10.1038/s41576-022-00483-8 PMC9028907 35459859

[2] B. Korber , W. M. Fischer , S. Gnanakaran , et al., “Tracking Changes in SARS‐CoV‐2 Spike: Evidence That D614G Increases Infectivity of the COVID‐19 Virus,” Cell 182, no. 4 (2020): 812–827. 32697968 10.1016/j.cell.2020.06.043 PMC7332439

[3] A. Telenti , E. B. Hodcroft , and D. L. Robertson , “The Evolution and Biology of SARS‐CoV‐2 Variants,” Cold Spring Harbor Perspectives in Medicine 12, no. 5 (2022): a041390. 35444005 10.1101/cshperspect.a041390 PMC9159258

[4] J. Hadfield , C. Megill , S. M. Bell , et al., “Nextstrain: Real‐Time Tracking of Pathogen Evolution,” Bioinformatics 34, no. 23 (2018): 4121–4123. 29790939 10.1093/bioinformatics/bty407 PMC6247931

[5] B. Berkhout and E. Herrera‐Carrillo , “SARS‐CoV‐2 Evolution: On the Sudden Appearance of the Omicron Variant,” Journal of Virology 96, no. 7 (2022): e0009022. 35293771 10.1128/jvi.00090-22 PMC9006888

[6] R. Viana , S. Moyo , D. G. Amoako , et al., “Rapid Epidemic Expansion of the SARS‐CoV‐2 Omicron Variant in Southern Africa,” Nature 603, no. 7902 (2022): 679–686. 35042229 10.1038/s41586-022-04411-y PMC8942855

[7] S. Poudel , A. Ishak , J. Perez‐Fernandez , et al., “Highly Mutated SARS‐CoV‐2 Omicron Variant Sparks Significant Concern Among Global Experts ‐ What is Known so far?,” Travel medicine and infectious disease 45 (2022): 102234. 34896326 10.1016/j.tmaid.2021.102234 PMC8666662

[8] J. L. H. Tsui , J. T. McCrone , B. Lambert , et al., “Genomic Assessment of Invasion Dynamics of SARS‐CoV‐2 Omicron BA.1,” Science 381, no. 6655 (2023): 336–343. 37471538 10.1126/science.adg6605 PMC10866301

[9] H. Tegally , M. Moir , J. Everatt , et al., “Emergence of SARS‐CoV‐2 Omicron Lineages BA.4 and BA.5 in South Africa,” Nature Medicine 28, no. 9 (2022): 1785–1790. 10.1038/s41591-022-01911-2 PMC9499863 35760080

[10] I. Bergeri , M. G. Whelan , H. Ware , et al., “Global SARS‐CoV‐2 Seroprevalence From January 2020 to April 2022: A Systematic Review and Meta‐Analysis of Standardized Population‐Based Studies,” PLoS Medicine 19, no. 11 (2022): e1004107. 36355774 10.1371/journal.pmed.1004107 PMC9648705

[11] R. Naeimi , M. Sepidarkish , A. Mollalo , et al., “SARS‐CoV‐2 Seroprevalence in Children Worldwide: A Systematic Review and Meta‐Analysis,” EClinicalMedicine 56 (2023): 101786. 36590788 10.1016/j.eclinm.2022.101786 PMC9795163

[12] L. Chen , Y. He , H. Liu , Y. Shang , and G. Guo , “Potential Immune Evasion of the Severe Acute Respiratory Syndrome Coronavirus 2 Omicron Variants,” Frontiers in Immunology 15 (2024): 1339660. 38464527 10.3389/fimmu.2024.1339660 PMC10924305

[13] R. Gili and R. Burioni , “SARS‐CoV‐2 Before and After Omicron: Two Different Viruses and Two Different Diseases?,” Journal of Translational Medicine 21, no. 1 (2023): 251. 37038133 10.1186/s12967-023-04095-6 PMC10088248

[14] K. Guo , B. S. Barrett , K. L. Mickens , et al., “Interferon Resistance of Emerging SARS‐CoV‐2 Variants,” bioRxiv, 2021, 2021.03.20.436257. 10.1073/pnas.2203760119 PMC9371743 35867811

[15] M. Le Page , “Understanding Omicron,” New Scientist 253, no. 3369 (2022): 8–9. 10.1016/S0262-4079(22)00030-6 PMC8759760 35068646

[16] J. Liu , Q. He , F. Gao , et al., “Heterologous Omicron‐Adapted Vaccine as a Secondary Booster Promotes Neutralizing Antibodies Against Omicron and its Sub‐Lineages in Mice,” Emerging Microbes & Infections 12, no. 1 (2023): e2143283. 36377297 10.1080/22221751.2022.2143283 PMC9754032

[17] S. Farheen , Y. Araf , Y. D. Tang , and C. Zheng , “The Deltacron Conundrum: Its Origin and Potential Health Risks,” Journal of Medical Virology 94, no. 11 (2022): 5096–5102. 35815524 10.1002/jmv.27990

[18] D. V. Parums , “Editorial: The XBB.1.5 (‘Kraken’) Subvariant of Omicron SARS‐CoV‐2 and its Rapid Global Spread,” Medical Science Monitor 29 (2023): e939580. 36722047 10.12659/MSM.939580 PMC9901170

[19] R. Link‐Gelles , A. A. Ciesla , L. E. Roper , et al., “Early Estimates of Bivalent mRNA Booster Dose Vaccine Effectiveness in Preventing Symptomatic SARS‐CoV‐2 Infection Attributable to Omicron BA.5‐ and XBB/XBB.1.5‐Related Sublineages Among Immunocompetent Adults—Increasing Community Access to Testing Program, United States, December 2022‐January 2023,” Morbidity and Mortality Weekly Report 72, no. 5 (2023): 119–124. 36730051 10.15585/mmwr.mm7205e1 PMC9927070

[20] A. Lai , A. Bergna , C. Della Ventura , et al., “Epidemiological and Clinical Features of SARS‐CoV‐2 Variants Circulating Between April‐December 2021 in Italy,” Viruses 14, no. 11 (2022): 2508. 36423117 10.3390/v14112508 PMC9699621

[21] B. Q. Minh , H. A. Schmidt , O. Chernomor , et al., “IQ‐TREE 2: New Models and Efficient Methods for Phylogenetic Inference in the Genomic Era,” Molecular Biology and Evolution 37, no. 5 (2020): 1530–1534. 32011700 10.1093/molbev/msaa015 PMC7182206

[22] R. Bouckaert , T. G. Vaughan , J. Barido‐Sottani , et al., “BEAST 2.5: An Advanced Software Platform for Bayesian Evolutionary Analysis,” PLoS Computational Biology 15, no. 4 (2019): e1006650. 30958812 10.1371/journal.pcbi.1006650 PMC6472827

[23] A. Bergna , A. Lai , C. D. Ventura , et al., “Genomic Epidemiology of the Main SARS‐CoV‐2 Variants in Italy Between Summer 2020 and Winter 2021,” Journal of Medical Virology 95, no. 11 (2023): e29193. 37927140 10.1002/jmv.29193

[24] A. Lai , A. Bergna , S. Toppo , et al., “Phylogeography and Genomic Epidemiology of SARS‐CoV‐2 in Italy and Europe With Newly Characterized Italian Genomes Between February–June 2020,” Scientific Reports 12, no. 1 (2022): 5736. 35388091 10.1038/s41598-022-09738-0 PMC8986836

[25] A. Rambaut , A. J. Drummond , D. Xie , G. Baele , and M. A. Suchard , “Posterior Summarization in Bayesian Phylogenetics Using Tracer 1.7,” Systematic Biology 67, no. 5 (2018): 901–904. 29718447 10.1093/sysbio/syy032 PMC6101584

[26] T. Stadler , D. Kühnert , S. Bonhoeffer , and A. J. Drummond , “Birth‐Death Skyline Plot Reveals Temporal Changes of Epidemic Spread in HIV and Hepatitis C Virus (HCV),” Proceedings of the National Academy of Sciences 110, no. 1 (2013): 228–233. 10.1073/pnas.1207965110 PMC3538216 23248286

[27] Q. Li , X. Guan , P. Wu , et al., “Early Transmission Dynamics in Wuhan, China, of Novel Coronavirus‐Infected Pneumonia,” New England Journal of Medicine 382, no. 13 (2020): 1199–1207. 31995857 10.1056/NEJMoa2001316 PMC7121484

[28] P. R. Walker , O. G. Pybus , A. Rambaut , and E. C. Holmes , “Comparative Population Dynamics of HIV‐1 Subtypes B and C: Subtype‐Specific Differences in Patterns of Epidemic Growth,” Infection, Genetics and Evolution 5, no. 3 (2005): 199–208. 10.1016/j.meegid.2004.06.011 15737910

[29] A. M. Carabelli , T. P. Peacock , L. G. Thorne , et al., “SARS‐CoV‐2 Variant Biology: Immune Escape, Transmission and Fitness,” Nature Reviews Microbiology 21, no. 3 (2023): 162–177. 36653446 10.1038/s41579-022-00841-7 PMC9847462

[30] C. Roemer , D. J. Sheward , R. Hisner , et al., “SARS‐CoV‐2 Evolution in the Omicron Era,” Nature Microbiology 8, no. 11 (2023): 1952–1959. 10.1038/s41564-023-01504-w 37845314

[31] H. Tegally , E. Wilkinson , J. L. H. Tsui , et al., “Dispersal Patterns and Influence of Air Travel During the Global Expansion of SARS‐CoV‐2 Variants of Concern,” Cell 186, no. 15 (2023): 3277–3290. 37413988 10.1016/j.cell.2023.06.001 PMC10247138

[32] V. Chavda , R. Bezbaruah , K. Deka , L. Nongrang , and T. Kalita , “The Delta and Omicron Variants of SARS‐CoV‐2: What We Know So Far,” Vaccines 10, no. 11 (2022): 1926. 36423021 10.3390/vaccines10111926 PMC9698608

[33] N. Radhakrishnan , M. Liu , B. Idowu , et al., “Comparison of the Clinical Characteristics of SARS‐CoV‐2 Delta (B.1.617.2) and Omicron (B.1.1.529) Infected Patients From a Single Hospitalist Service,” BMC Infectious Diseases 23, no. 1 (2023): 747. 37907849 10.1186/s12879-023-08714-x PMC10617227

[34] N. Van Goethem , P. Y. J. Chung , M. Meurisse , et al., “Clinical Severity of SARS‐CoV‐2 Omicron Variant Compared With Delta Among Hospitalized COVID‐19 Patients in Belgium During Autumn and Winter Season 2021‐2022,” Viruses 14, no. 6 (2022): 1297. 35746768 10.3390/v14061297 PMC9227815

[35] A. X. Han , E. Kozanli , J. Koopsen , et al., “Regional Importation and Asymmetric Within‐Country Spread of SARS‐CoV‐2 Variants of Concern in the Netherlands,” eLife 11 (2022): e78770. 36097810 10.7554/eLife.78770 PMC9470152

[36] A. J. Kucharski , M. Jit , J. G. Logan , et al., “Travel Measures in the SARS‐CoV‐2 Variant Era Need Clear Objectives,” Lancet 399, no. 10333 (2022): 1367–1369. 35247312 10.1016/S0140-6736(22)00366-X PMC8890754

[37] Y. Wang , Y. Long , F. Wang , C. Li , and W. Liu , “Characterization of SARS‐CoV‐2 Recombinants and Emerging Omicron Sublineages,” International Journal of Medical Sciences 20, no. 1 (2023): 151–162. 36619228 10.7150/ijms.79116 PMC9812801

[38] M. Francesconi , M. Giovanetti , L. De Florio , et al., “Genomic Epidemiology Unveil the Omicron Transmission Dynamics in Rome, Italy,” Pathogens 11, no. 9 (2022): 1011. 36145443 10.3390/pathogens11091011 PMC9505927

[39] M. Ragonnet‐Cronin , E. Hodcroft , S. Hué , et al., “Automated Analysis of Phylogenetic Clusters,” BMC Bioinformatics 14 (2013): 317. 24191891 10.1186/1471-2105-14-317 PMC4228337

[40] R. Xie , K. M. Edwards , D. C. Adam , et al., “Resurgence of Omicron BA.2 in SARS‐CoV‐2 Infection‐Naive Hong Kong,” Nature Communications 14, no. 1 (2023): 2422. 10.1038/s41467-023-38201-5 PMC10134727 37105966

[41] F. P. Lyngse , C. T. Kirkeby , M. Denwood , et al., “Household Transmission of SARS‐CoV‐2 Omicron Variant of Concern Subvariants BA.1 and BA.2 in Denmark,” Nature Communications 13, no. 1 (2022): 5760. 10.1038/s41467-022-33498-0 PMC9524324 36180438

[42] Y. Zhou , H. Zhi , and Y. Teng , “The Outbreak of SARS‐CoV‐2 Omicron Lineages, Immune Escape, and Vaccine Effectivity,” Journal of Medical Virology 95, no. 1 (2023): e28138. 36097349 10.1002/jmv.28138 PMC9538491

