# Supplementary material for: Genomic Epidemiology of the Main SARS‐CoV‐2 Variants Circulating in Italy During the Omicron Era
Source: J Med Virol. 2025 Feb 12;97(2):e70215. doi: 10.1002/jmv.70215 (PMC11816846; doi:10.1002/jmv.70215)
Supplement: Supplementary file 1 — Supporting information. [file JMV-97-e70215-s001.docx]

**Supporting Information**

Table S1. Italian and International datasets composition.

|  |  |  |  | **International dataset** | | |
| --- | --- | --- | --- | --- | --- | --- |
|  |  | **Italian dataset** | | |  |  |
|  | variants | SCIRE Sequences | GISAID Sequences | Total | GISAID not Italian sequences | Total |
| Omicron | **BA.1** | 268 | 612 | **880** | 957 | **1,837** |
|  | **BA.2** | 677 | 950 | **1,627** | 1,619 | **3,246** |
|  | **BA.5** | 713 | 1,048 | **1,761** | 1,458 | **3,219** |

Table S2. Distribution in terms of region of Italian sequences included in the datasets.

| **Regions** | **BA.1 (n=880)** | **BA.2 (n=1,627)** | **BA.2 (n=1,761)** |
| --- | --- | --- | --- |
| Abruzzo | 39 | 46 | 72 |
| Basilicata | 8 | 22 | 14 |
| Calabria | 13 | 25 | 17 |
| Campania | 48 | 73 | 73 |
| Emilia-Romagna | 50 | 73 | 77 |
| Friuli-Venezia Giulia | 40 | 64 | 77 |
| Lazio | 105 | 148 | 106 |
| Liguria | 119 | 253 | 184 |
| Lombardy | 164 | 355 | 426 |
| Marche | 10 | 98 | 158 |
| Molise | / | / | / |
| Piedmonte | 40 | 63 | 59 |
| Apulia | 37 | 65 | 71 |
| Sardinia | / | 5 | 9 |
| Sicily | 50 | 74 | 77 |
| Tuscany | 13 | 50 | 60 |
| Trentino Alto Adige | 40 | 59 | 96 |
| Umbria | 49 | 78 | 101 |
| Aosta Valley | 15 | 7 | 9 |
| Veneto | 40 | 69 | 75 |

Table S3. Lineages’ distribution in the SCIRE studied cohort.

|  |  | **January** | **February** | **March** | **April** | **May** | **June** | **July** | **August** | **September** | **October** | **November** | **December** |
| --- | --- | --- | --- | --- | --- | --- | --- | --- | --- | --- | --- | --- | --- |
|  | **lineage** | **2482** | **953** | **1100** | **911** | **624** | **477** | **529** | **396** | **397** | **345** | **389** | **367** |
| **B.1** | B.1 | 1 | 0 | 0 | 0 | 0 | 0 | 0 | 0 | 0 | 0 | 0 | 0 |
|  | B.1.177 | 0 | 0 | 0 | 0 | 0 | 0 | 0 | 0 | 0 | 1 | 0 | 0 |
|  | B.1.1.530 | 0 | 1 | 0 | 0 | 0 | 1 | 0 | 0 | 0 | 0 | 0 | 0 |
|  | B.1.1.531 | 0 | 1 | 0 | 0 | 0 | 1 | 0 | 0 | 0 | 0 | 0 | 0 |
|  | B.1.1.532 | 0 | 1 | 0 | 0 | 0 | 1 | 0 | 0 | 0 | 0 | 0 | 0 |
| **Delta** | B.1.617.2 | 86 | 2 | 0 | 0 | 0 | 0 | 0 | 0 | 0 | 0 | 0 | 0 |
|  | AY.116 | 1 | 0 | 0 | 0 | 0 | 0 | 0 | 0 | 0 | 0 | 0 | 0 |
|  | AY.122 | 0 | 0 | 0 | 0 | 0 | 0 | 0 | 1 | 0 | 0 | 0 | 0 |
|  | AY.122.3 | 1 | 0 | 0 | 0 | 0 | 0 | 0 | 0 | 0 | 0 | 0 | 0 |
|  | AY.126 | 1 | 0 | 0 | 0 | 0 | 0 | 0 | 0 | 0 | 0 | 0 | 0 |
|  | AY.127 | 2 | 0 | 0 | 0 | 0 | 0 | 0 | 0 | 0 | 0 | 0 | 0 |
|  | AY.23 | 4 | 0 | 0 | 0 | 0 | 0 | 0 | 0 | 0 | 0 | 0 | 0 |
|  | AY.4 | 21 | 0 | 0 | 0 | 0 | 0 | 0 | 0 | 0 | 0 | 0 | 0 |
|  | AY.4.2 | 11 | 0 | 0 | 0 | 0 | 0 | 0 | 0 | 0 | 0 | 0 | 0 |
|  | AY.4.2.2 | 4 | 0 | 0 | 0 | 0 | 0 | 0 | 0 | 0 | 0 | 0 | 0 |
|  | AY.4.2.3 | 2 | 0 | 0 | 0 | 0 | 0 | 0 | 0 | 0 | 0 | 0 | 0 |
|  | AY.4.5 | 1 | 0 | 0 | 0 | 0 | 0 | 0 | 0 | 0 | 0 | 0 | 0 |
|  | AY.43 | 22 | 0 | 0 | 0 | 0 | 0 | 0 | 0 | 0 | 0 | 0 | 0 |
|  | AY.44 | 1 | 0 | 0 | 0 | 0 | 0 | 0 | 0 | 0 | 0 | 0 | 0 |
|  | AY.5 | 1 | 0 | 0 | 0 | 0 | 0 | 0 | 0 | 0 | 0 | 0 | 0 |
| **BA.1** | BA.1 | 2011 | 786 | 518 | 11 | 2 | 2 | 77 | 5 | 1 | 1 | 0 | 0 |
|  | BA.1.1 | 113 | 33 | 66 | 15 | 6 | 2 | 5 | 1 | 0 | 0 | 0 | 0 |
|  | BA.1.1.1 | 1 | 2 | 7 | 2 | 1 | 0 | 0 | 0 | 0 | 0 | 0 | 0 |
|  | BA.1.1.10 | 0 | 0 | 1 | 1 | 0 | 0 | 0 | 0 | 0 | 0 | 0 | 0 |
|  | BA.1.1.11 | 8 | 2 | 1 | 0 | 0 | 1 | 0 | 0 | 0 | 0 | 0 | 0 |
|  | BA.1.1.14 | 0 | 0 | 4 | 1 | 0 | 0 | 1 | 0 | 0 | 0 | 0 | 0 |
|  | BA.1.1.15 | 2 | 0 | 0 | 0 | 0 | 0 | 0 | 0 | 0 | 0 | 0 | 0 |
|  | BA.1.1.18 | 0 | 0 | 2 | 0 | 0 | 0 | 0 | 0 | 0 | 0 | 0 | 0 |
|  | BA.1.1.16 | 14 | 1 | 0 | 3 | 0 | 0 | 0 | 0 | 0 | 0 | 0 | 0 |
|  | BA.1.11 | 0 | 5 | 0 | 0 | 0 | 0 | 0 | 0 | 0 | 0 | 0 | 0 |
|  | BA.1.15 | 6 | 5 | 0 | 1 | 0 | 0 | 0 | 0 | 0 | 0 | 0 | 0 |
|  | BA.1.15.1 | 0 | 0 | 1 | 0 | 0 | 0 | 0 | 0 | 0 | 0 | 0 | 0 |
|  | BA.1.16 | 1 | 0 | 1 | 0 | 0 | 0 | 0 | 0 | 0 | 0 | 0 | 0 |
|  | BA.1.17 | 7 | 4 | 1 | 0 | 0 | 0 | 1 | 0 | 0 | 0 | 0 | 0 |
|  | BA.1.17.2 | 76 | 12 | 20 | 10 | 9 | 0 | 0 | 0 | 0 | 0 | 0 | 0 |
|  | BA.1.18 | 1 | 0 | 1 | 0 | 0 | 0 | 0 | 0 | 0 | 0 | 0 | 0 |
|  | BA.1.20 | 0 | 1 | 2 | 0 | 0 | 0 | 0 | 0 | 0 | 0 | 0 | 0 |
|  | BA.1.21 | 0 | 1 | 1 | 0 | 1 | 0 | 0 | 0 | 0 | 0 | 0 | 0 |
|  | BA.1.21.1 | 1 | 1 | 2 | 0 | 0 | 0 | 0 | 0 | 0 | 0 | 0 | 0 |
|  | BA.1.3 | 0 | 1 | 0 | 0 | 0 | 0 | 0 | 0 | 0 | 0 | 0 | 0 |
|  | BA.1.5 | 0 | 2 | 0 | 0 | 0 | 0 | 0 | 0 | 0 | 0 | 0 | 0 |
|  | BA.1.7 | 0 | 2 | 0 | 0 | 0 | 0 | 0 | 0 | 0 | 0 | 0 | 0 |
|  | BA.1.7.2 | 0 | 11 | 0 | 0 | 0 | 0 | 0 | 0 | 0 | 0 | 0 | 0 |
| **BA.2** | BA.2 | 51 | 58 | 407 | 828 | 566 | 215 | 16 | 5 | 5 | 1 | 7 | 2 |
|  | BA.2.1 | 0 | 0 | 0 | 0 | 0 | 0 | 0 | 1 | 0 | 0 | 0 | 0 |
|  | BA.2.3 | 0 | 0 | 13 | 6 | 3 | 2 | 0 | 0 | 0 | 0 | 0 | 0 |
|  | BA.2.3.6 | 0 | 0 | 2 | 0 | 0 | 0 | 0 | 0 | 0 | 0 | 0 | 0 |
|  | BA.2.3.7 | 0 | 0 | 0 | 0 | 1 | 0 | 0 | 0 | 0 | 0 | 0 | 0 |
|  | BA.2.3.9 | 0 | 0 | 1 | 0 | 0 | 0 | 0 | 0 | 0 | 0 | 0 | 0 |
|  | BA.2.3.15 | 0 | 1 | 3 | 3 | 7 | 1 | 0 | 0 | 0 | 0 | 0 | 0 |
|  | BA.2.3.20 | 0 | 0 | 0 | 0 | 0 | 0 | 0 | 0 | 0 | 1 | 1 | 0 |
|  | BA.2.5 | 0 | 0 | 0 | 1 | 0 | 0 | 0 | 0 | 0 | 0 | 0 | 0 |
|  | BA.2.9 | 2 | 8 | 29 | 21 | 9 | 4 | 0 | 0 | 0 | 0 | 0 | 0 |
|  | BA.2.10 | 0 | 0 | 7 | 5 | 0 | 0 | 0 | 0 | 0 | 0 | 0 | 0 |
|  | BA.2.10.1 | 0 | 0 | 2 | 0 | 0 | 0 | 0 | 0 | 0 | 0 | 0 | 0 |
|  | BA.2.11 | 0 | 0 | 2 | 0 | 0 | 0 | 0 | 0 | 0 | 0 | 0 | 0 |
|  | BA.2.12 | 0 | 0 | 0 | 0 | 1 | 0 | 1 | 0 | 0 | 0 | 0 | 0 |
|  | BA.2.12.1 | 0 | 0 | 0 | 1 | 2 | 5 | 5 | 1 | 0 | 0 | 0 | 0 |
|  | BA.2.13 | 0 | 0 | 0 | 0 | 0 | 0 | 1 | 0 | 0 | 0 | 0 | 0 |
|  | BA.2.18 | 0 | 0 | 0 | 0 | 0 | 1 | 0 | 0 | 0 | 0 | 0 | 0 |
|  | BA.2.22 | 0 | 0 | 0 | 1 | 2 | 0 | 0 | 0 | 0 | 0 | 0 | 0 |
|  | BA.2.23 | 0 | 0 | 0 | 0 | 0 | 1 | 0 | 0 | 0 | 0 | 0 | 0 |
|  | BA.2.32 | 0 | 0 | 0 | 0 | 1 | 0 | 0 | 0 | 0 | 0 | 0 | 0 |
|  | BA.2.36 | 0 | 0 | 1 | 0 | 2 | 0 | 0 | 1 | 0 | 0 | 0 | 0 |
|  | BA.2.37 | 0 | 0 | 1 | 0 | 0 | 0 | 0 | 0 | 0 | 0 | 0 | 0 |
|  | BA.2.44 | 0 | 0 | 0 | 0 | 0 | 1 | 0 | 0 | 0 | 0 | 0 | 0 |
|  | BA.2.52 | 0 | 0 | 0 | 0 | 0 | 1 | 0 | 0 | 0 | 0 | 0 | 0 |
|  | BA.2.54 | 0 | 0 | 0 | 0 | 0 | 0 | 1 | 0 | 0 | 0 | 0 | 0 |
|  | BA.2.56 | 0 | 0 | 0 | 0 | 0 | 1 | 1 | 0 | 0 | 0 | 0 | 0 |
|  | BA.2.65 | 0 | 0 | 1 | 1 | 1 | 0 | 0 | 0 | 0 | 0 | 0 | 0 |
|  | BA.2.75 | 0 | 0 | 0 | 0 | 0 | 0 | 0 | 1 | 0 | 0 | 0 | 0 |
|  | BA.2.75.2 | 0 | 0 | 0 | 0 | 0 | 0 | 0 | 0 | 0 | 1 | 0 | 1 |
|  | BA.2.75.5 | 0 | 0 | 0 | 0 | 0 | 0 | 0 | 0 | 1 | 1 | 0 | 0 |
| **BA.4** | BA.4 | 0 | 0 | 0 | 0 | 8 | 83 | 9 | 6 | 0 | 0 | 1 | 0 |
|  | BA.4.1 | 0 | 0 | 0 | 0 | 0 | 2 | 7 | 2 | 0 | 0 | 0 | 0 |
|  | BA.4.1.1 | 0 | 0 | 0 | 0 | 0 | 0 | 0 | 0 | 0 | 1 | 0 | 0 |
|  | BA.4.1.8 | 0 | 0 | 0 | 0 | 0 | 0 | 0 | 0 | 0 | 1 | 0 | 0 |
|  | BA.4.3 | 0 | 0 | 0 | 0 | 0 | 7 | 9 | 3 | 0 | 0 | 0 | 0 |
|  | BA.4.6 | 0 | 0 | 0 | 0 | 0 | 0 | 4 | 4 | 4 | 2 | 2 | 2 |
|  | BA.4.7 | 0 | 0 | 0 | 0 | 0 | 0 | 0 | 2 | 1 | 0 | 0 | 0 |
| **BA.5** | BA.5 | 0 | 0 | 0 | 0 | 2 | 84 | 243 | 189 | 174 | 88 | 95 | 73 |
|  | BA.5.1 | 0 | 0 | 0 | 0 | 0 | 13 | 42 | 34 | 26 | 19 | 11 | 4 |
|  | BA.5.1.1 | 0 | 0 | 0 | 0 | 0 | 1 | 1 | 2 | 0 | 0 | 0 | 0 |
|  | BA.5.1.2 | 0 | 0 | 0 | 0 | 0 | 0 | 0 | 2 | 0 | 1 | 0 | 0 |
|  | BA.5.1.3 | 0 | 0 | 0 | 0 | 0 | 0 | 0 | 1 | 1 | 0 | 0 | 0 |
|  | BA.5.1.5 | 0 | 0 | 0 | 0 | 0 | 0 | 0 | 0 | 6 | 2 | 4 | 0 |
|  | BA.5.1.8 | 0 | 0 | 0 | 0 | 0 | 0 | 1 | 1 | 1 | 0 | 0 | 0 |
|  | BA.5.1.9 | 0 | 0 | 0 | 0 | 0 | 0 | 0 | 0 | 0 | 1 | 0 | 0 |
|  | BA.5.1.10 | 0 | 0 | 0 | 0 | 0 | 1 | 6 | 10 | 6 | 1 | 2 | 0 |
|  | BA.5.1.12 | 0 | 0 | 0 | 0 | 0 | 0 | 0 | 0 | 0 | 1 | 0 | 0 |
|  | BA.5.1.18 | 0 | 0 | 0 | 0 | 0 | 0 | 0 | 0 | 0 | 1 | 2 | 0 |
|  | BA.5.1.19 | 0 | 0 | 0 | 0 | 0 | 0 | 0 | 0 | 1 | 0 | 0 | 0 |
|  | BA.5.1.21 | 0 | 0 | 0 | 0 | 0 | 0 | 1 | 0 | 0 | 0 | 0 | 0 |
|  | BA.5.1.22 | 0 | 0 | 0 | 0 | 0 | 0 | 1 | 2 | 0 | 0 | 0 | 0 |
|  | BA.5.1.23 | 0 | 0 | 0 | 0 | 0 | 0 | 0 | 0 | 1 | 3 | 2 | 0 |
|  | BA.5.1.24 | 0 | 0 | 0 | 0 | 0 | 0 | 0 | 1 | 1 | 0 | 0 | 0 |
|  | BA.5.1.28 | 0 | 0 | 0 | 0 | 0 | 0 | 0 | 0 | 0 | 1 | 0 | 0 |
|  | BA.5.1.30 | 0 | 0 | 0 | 0 | 0 | 0 | 0 | 3 | 1 | 1 | 0 | 0 |
|  | BA.5.2 | 1 | 0 | 2 | 0 | 0 | 28 | 49 | 44 | 59 | 55 | 16 | 1 |
|  | BA.5.2.1 | 0 | 0 | 0 | 0 | 0 | 6 | 14 | 35 | 33 | 32 | 8 | 1 |
|  | BA.5.2.2 | 0 | 0 | 0 | 0 | 0 | 1 | 0 | 0 | 0 | 0 | 0 | 0 |
|  | BA.5.2.3 | 0 | 0 | 0 | 0 | 0 | 0 | 0 | 2 | 0 | 2 | 0 | 0 |
|  | BA.5.2.4 | 0 | 0 | 0 | 0 | 0 | 0 | 0 | 0 | 1 | 0 | 0 | 0 |
|  | BA.5.2.6 | 0 | 0 | 0 | 0 | 0 | 0 | 0 | 0 | 0 | 3 | 6 | 1 |
|  | BA.5.2.7 | 0 | 0 | 0 | 0 | 0 | 0 | 0 | 0 | 0 | 1 | 0 | 0 |
|  | BA.5.2.8 | 0 | 0 | 0 | 0 | 0 | 0 | 0 | 0 | 0 | 1 | 0 | 0 |
|  | BA.5.2.9 | 0 | 0 | 0 | 0 | 0 | 0 | 0 | 0 | 3 | 0 | 2 | 0 |
|  | BA.5.2.14 | 0 | 0 | 0 | 0 | 0 | 0 | 0 | 0 | 0 | 1 | 2 | 0 |
|  | BA.5.2.16 | 0 | 0 | 0 | 0 | 0 | 0 | 0 | 1 | 0 | 1 | 0 | 0 |
|  | BA.5.2.18 | 0 | 0 | 0 | 0 | 0 | 0 | 0 | 0 | 0 | 1 | 3 | 1 |
|  | BA.5.2.19 | 0 | 0 | 0 | 0 | 0 | 0 | 0 | 0 | 0 | 1 | 0 | 0 |
|  | BA.5.2.20 | 0 | 0 | 0 | 0 | 0 | 0 | 0 | 2 | 4 | 2 | 3 | 1 |
|  | BA.5.2.21 | 0 | 0 | 0 | 0 | 0 | 0 | 1 | 1 | 1 | 2 | 1 | 0 |
|  | BA.5.2.22 | 0 | 0 | 0 | 0 | 0 | 0 | 0 | 0 | 0 | 1 | 1 | 0 |
|  | BA.5.2.25 | 0 | 0 | 0 | 0 | 0 | 0 | 0 | 0 | 0 | 3 | 0 | 0 |
|  | BA.5.2.26 | 0 | 0 | 0 | 0 | 0 | 0 | 0 | 0 | 1 | 0 | 0 | 0 |
|  | BA.5.2.28 | 0 | 0 | 0 | 0 | 0 | 0 | 0 | 0 | 0 | 0 | 1 | 0 |
|  | BA.5.2.33 | 0 | 0 | 0 | 0 | 0 | 0 | 0 | 0 | 1 | 0 | 0 | 0 |
|  | BA.5.2.34 | 0 | 0 | 0 | 0 | 0 | 0 | 0 | 0 | 0 | 0 | 1 | 0 |
|  | BA.5.2.35 | 0 | 0 | 0 | 0 | 0 | 0 | 0 | 0 | 0 | 0 | 0 | 1 |
|  | BA.5.2.47 | 0 | 0 | 0 | 0 | 0 | 0 | 0 | 0 | 0 | 0 | 0 | 2 |
|  | BA.5.2.59 | 0 | 0 | 0 | 0 | 0 | 0 | 0 | 0 | 1 | 2 | 0 | 0 |
|  | BA.5.2.62 | 0 | 0 | 0 | 0 | 0 | 0 | 0 | 0 | 1 | 0 | 0 | 0 |
|  | BA.5.3.1 | 0 | 0 | 0 | 0 | 0 | 1 | 3 | 0 | 4 | 3 | 1 | 3 |
|  | BA.5.5 | 0 | 0 | 0 | 0 | 0 | 0 | 0 | 0 | 3 | 1 | 0 | 0 |
|  | BA.5.6 | 0 | 0 | 0 | 0 | 0 | 0 | 0 | 0 | 1 | 1 | 0 | 0 |
|  | BA.5.9 | 0 | 1 | 0 | 0 | 0 | 0 | 0 | 0 | 2 | 2 | 4 | 0 |
|  | BA.5.28 | 0 | 0 | 0 | 0 | 0 | 0 | 0 | 0 | 0 | 1 | 0 | 0 |
| **BD.X** | BD.1 | 0 | 0 | 1 | 0 | 0 | 0 | 0 | 0 | 0 | 0 | 0 | 0 |
| **BE.X** | BE.1 | 0 | 0 | 0 | 0 | 0 | 2 | 14 | 7 | 6 | 3 | 1 | 0 |
|  | BE.1.1 | 0 | 0 | 0 | 0 | 0 | 2 | 6 | 3 | 4 | 13 | 3 | 0 |
|  | BE.1.1.1 | 0 | 0 | 0 | 0 | 0 | 0 | 0 | 0 | 0 | 1 | 7 | 4 |
|  | BE.1.1.2 | 0 | 0 | 0 | 0 | 0 | 0 | 0 | 0 | 1 | 1 | 2 | 0 |
|  | BE.1.2 | 0 | 0 | 0 | 0 | 0 | 0 | 0 | 0 | 0 | 0 | 1 | 0 |
|  | BE.1.3 | 0 | 0 | 0 | 0 | 0 | 0 | 1 | 0 | 0 | 0 | 0 | 0 |
|  | BE.4 | 0 | 0 | 0 | 0 | 0 | 0 | 0 | 0 | 0 | 1 | 0 | 0 |
| **BF.X** | BF.1 | 0 | 0 | 0 | 0 | 0 | 2 | 2 | 2 | 1 | 0 | 0 | 0 |
|  | BF.1.1 | 0 | 0 | 0 | 0 | 0 | 0 | 0 | 0 | 0 | 1 | 0 | 0 |
|  | BF.3 | 0 | 0 | 0 | 0 | 0 | 0 | 0 | 0 | 1 | 0 | 0 | 0 |
|  | BF.4 | 0 | 0 | 0 | 0 | 0 | 0 | 0 | 1 | 1 | 0 | 1 | 0 |
|  | BF.5 | 0 | 0 | 0 | 0 | 0 | 1 | 0 | 11 | 19 | 6 | 2 | 0 |
|  | BF.6 | 0 | 0 | 0 | 0 | 0 | 0 | 1 | 1 | 0 | 0 | 0 | 0 |
|  | BF.7 | 0 | 0 | 0 | 0 | 0 | 0 | 0 | 7 | 9 | 22 | 33 | 21 |
|  | BF.7.4 | 0 | 0 | 0 | 0 | 0 | 0 | 0 | 0 | 1 | 1 | 3 | 0 |
|  | BF.7.4.1 | 0 | 0 | 0 | 0 | 0 | 0 | 0 | 0 | 0 | 0 | 0 | 1 |
|  | BF.7.5 | 0 | 0 | 0 | 0 | 0 | 0 | 0 | 0 | 0 | 0 | 1 | 3 |
|  | BF.7.6 | 0 | 0 | 0 | 0 | 0 | 0 | 0 | 0 | 0 | 1 | 3 | 0 |
|  | BF.7.7 | 0 | 0 | 0 | 0 | 0 | 0 | 0 | 0 | 0 | 0 | 1 | 0 |
|  | BF.7.8 | 0 | 0 | 0 | 0 | 0 | 0 | 0 | 0 | 0 | 0 | 2 | 0 |
|  | BF.7.14 | 0 | 0 | 0 | 0 | 0 | 0 | 0 | 0 | 0 | 0 | 0 | 1 |
|  | BF.7.20 | 0 | 0 | 0 | 0 | 0 | 0 | 0 | 0 | 1 | 0 | 0 | 0 |
|  | BF.7.22 | 0 | 0 | 0 | 0 | 0 | 0 | 0 | 0 | 3 | 0 | 0 | 0 |
|  | BF.7.24 | 0 | 0 | 0 | 0 | 0 | 0 | 0 | 0 | 0 | 0 | 1 | 0 |
|  | BF.9 | 0 | 0 | 0 | 0 | 0 | 0 | 0 | 0 | 1 | 0 | 0 | 0 |
|  | BF.10 | 0 | 0 | 0 | 0 | 0 | 0 | 2 | 0 | 0 | 0 | 1 | 0 |
|  | BF.11 | 0 | 0 | 0 | 0 | 0 | 0 | 0 | 0 | 0 | 3 | 2 | 0 |
|  | BF.14 | 0 | 0 | 0 | 0 | 0 | 0 | 0 | 0 | 1 | 3 | 4 | 5 |
|  | BF.17 | 0 | 0 | 0 | 0 | 0 | 0 | 0 | 0 | 0 | 0 | 0 | 1 |
|  | BF.18 | 0 | 0 | 0 | 0 | 0 | 0 | 0 | 1 | 0 | 0 | 0 | 0 |
|  | BF.21 | 0 | 0 | 0 | 0 | 0 | 0 | 0 | 0 | 1 | 0 | 0 | 0 |
|  | BF.28 | 0 | 0 | 0 | 0 | 0 | 0 | 0 | 0 | 0 | 1 | 0 | 0 |
|  | BF.36 | 0 | 0 | 0 | 0 | 0 | 0 | 3 | 0 | 0 | 0 | 0 | 0 |
|  | BF.40 | 0 | 0 | 0 | 0 | 0 | 0 | 0 | 0 | 0 | 1 | 1 | 0 |
| **BL.X** | BL.1 | 0 | 0 | 0 | 0 | 0 | 0 | 0 | 0 | 0 | 0 | 0 | 3 |
| **BM.X** | BM.1.1.3 | 0 | 0 | 0 | 0 | 0 | 0 | 0 | 0 | 0 | 0 | 2 | 0 |
|  | BM.4.1.1 | 0 | 0 | 0 | 0 | 0 | 0 | 0 | 0 | 0 | 3 | 0 | 0 |
| **BN.X** | BN.1 | 0 | 0 | 0 | 0 | 0 | 0 | 0 | 0 | 0 | 2 | 2 | 0 |
|  | BN.1.2 | 0 | 0 | 0 | 0 | 0 | 0 | 0 | 0 | 0 | 0 | 0 | 4 |
|  | BN.1.2.1 | 0 | 0 | 0 | 0 | 0 | 0 | 0 | 0 | 0 | 0 | 0 | 1 |
|  | BN.1.3 | 0 | 0 | 0 | 0 | 0 | 0 | 0 | 0 | 0 | 1 | 5 | 8 |
|  | BN.1.3.1 | 0 | 0 | 0 | 0 | 0 | 0 | 0 | 0 | 0 | 0 | 0 | 1 |
|  | BN.1.4 | 0 | 0 | 0 | 0 | 0 | 0 | 0 | 0 | 0 | 1 | 0 | 0 |
|  | BN.1.5 | 0 | 0 | 0 | 0 | 0 | 0 | 0 | 0 | 0 | 0 | 1 | 1 |
| **CA.X** | CA.7 | 0 | 0 | 0 | 0 | 0 | 0 | 0 | 0 | 0 | 0 | 0 | 2 |
| **CH.X** | CH.1.1 | 0 | 0 | 0 | 0 | 0 | 0 | 0 | 0 | 0 | 0 | 0 | 3 |
| **CJ.X** | CJ.1.2 | 0 | 0 | 0 | 0 | 0 | 0 | 0 | 0 | 0 | 0 | 1 | 1 |
| **CK.X** | CK.1 | 0 | 0 | 0 | 0 | 0 | 0 | 0 | 0 | 0 | 2 | 1 | 0 |
|  | CK.2.1.1 | 0 | 0 | 0 | 0 | 0 | 0 | 0 | 0 | 0 | 1 | 1 | 3 |
| **CN.1** | CN.1 | 0 | 0 | 0 | 0 | 0 | 0 | 0 | 0 | 1 | 1 | 0 | 1 |
| **CL.X** | CL.1.3 | 0 | 0 | 0 | 0 | 0 | 0 | 0 | 0 | 0 | 0 | 0 | 1 |
| **CM.X** | CM.4 | 0 | 0 | 0 | 0 | 0 | 0 | 0 | 0 | 0 | 0 | 0 | 1 |
|  | CM.6.1 | 0 | 0 | 0 | 0 | 0 | 0 | 0 | 0 | 0 | 0 | 0 | 2 |
| **CP.X** | CP.1 | 0 | 0 | 0 | 0 | 0 | 0 | 0 | 0 | 0 | 0 | 0 | 3 |
| **EF.X** | EF.1 | 0 | 0 | 0 | 0 | 0 | 0 | 0 | 0 | 0 | 0 | 1 | 2 |
|  | EF.1.1 | 0 | 0 | 0 | 0 | 0 | 0 | 0 | 0 | 0 | 0 | 0 | 1 |
| **Recombinants** | XAZ | 0 | 0 | 0 | 0 | 0 | 0 | 0 | 0 | 0 | 1 | 0 | 0 |
|  | XC | 28 | 11 | 0 | 0 | 0 | 0 | 0 | 0 | 0 | 0 | 0 | 0 |
|  | XQ | 0 | 0 | 0 | 0 | 0 | 1 | 0 | 0 | 0 | 0 | 0 | 0 |
|  | XT | 0 | 0 | 0 | 0 | 0 | 2 | 0 | 0 | 0 | 0 | 0 | 0 |
|  | XBF | 0 | 0 | 0 | 0 | 0 | 0 | 0 | 0 | 0 | 0 | 1 | 0 |
|  | XBG | 0 | 0 | 0 | 0 | 0 | 0 | 0 | 0 | 0 | 0 | 0 | 1 |
|  | XBB.1 | 0 | 0 | 0 | 0 | 0 | 0 | 0 | 0 | 0 | 0 | 6 | 4 |
|  | XBB.1.4 | 0 | 0 | 0 | 0 | 0 | 0 | 0 | 0 | 0 | 0 | 3 | 0 |
|  | XBB.1.29 | 0 | 0 | 0 | 0 | 0 | 0 | 0 | 0 | 0 | 0 | 1 | 0 |
|  | XBB.2 | 0 | 0 | 0 | 0 | 0 | 0 | 0 | 0 | 0 | 0 | 0 | 2 |
| **BQ.1** | BQ.1 | 0 | 0 | 0 | 0 | 0 | 0 | 0 | 0 | 0 | 7 | 55 | 52 |
|  | BQ.1.1 | 0 | 0 | 0 | 0 | 0 | 0 | 0 | 0 | 1 | 19 | 48 | 95 |
|  | BQ.1.1.2 | 0 | 0 | 0 | 0 | 0 | 0 | 0 | 0 | 0 | 0 | 0 | 1 |
|  | BQ.1.1.3 | 0 | 0 | 0 | 0 | 0 | 0 | 0 | 0 | 0 | 2 | 0 | 4 |
|  | BQ.1.1.4 | 0 | 0 | 0 | 0 | 0 | 0 | 0 | 0 | 0 | 0 | 0 | 4 |
|  | BQ.1.1.7 | 0 | 0 | 0 | 0 | 0 | 0 | 0 | 0 | 0 | 0 | 1 | 0 |
|  | BQ.1.1.13 | 0 | 0 | 0 | 0 | 0 | 0 | 0 | 0 | 0 | 0 | 0 | 1 |
|  | BQ.1.1.15 | 0 | 0 | 0 | 0 | 0 | 0 | 0 | 0 | 0 | 0 | 0 | 2 |
|  | BQ.1.10 | 0 | 0 | 0 | 0 | 0 | 0 | 0 | 0 | 0 | 0 | 2 | 1 |
|  | BQ.1.10.1 | 0 | 0 | 0 | 0 | 0 | 0 | 0 | 0 | 0 | 0 | 0 | 2 |
|  | BQ.1.13 | 0 | 0 | 0 | 0 | 0 | 0 | 0 | 0 | 0 | 0 | 1 | 0 |
|  | BQ.1.1.18 | 0 | 0 | 0 | 0 | 0 | 0 | 0 | 0 | 0 | 0 | 1 | 5 |
|  | BQ.1.1.23 | 0 | 0 | 0 | 0 | 0 | 0 | 0 | 0 | 0 | 0 | 0 | 1 |
|  | BQ.1.1.28 | 0 | 0 | 0 | 0 | 0 | 0 | 0 | 0 | 0 | 0 | 0 | 1 |
|  | BQ.1.1.30 | 0 | 0 | 0 | 0 | 0 | 0 | 0 | 0 | 0 | 0 | 2 | 0 |
|  | BQ.1.1.31 | 0 | 0 | 0 | 0 | 0 | 0 | 0 | 0 | 0 | 0 | 0 | 1 |
|  | BQ.1.1.35 | 0 | 0 | 0 | 0 | 0 | 0 | 0 | 0 | 0 | 0 | 0 | 1 |
|  | BQ.1.1.47 | 0 | 0 | 0 | 0 | 0 | 0 | 0 | 0 | 0 | 0 | 0 | 8 |
|  | BQ.1.2 | 0 | 0 | 0 | 0 | 0 | 0 | 0 | 0 | 0 | 0 | 1 | 0 |
|  | BQ.1.15 | 0 | 0 | 0 | 0 | 0 | 0 | 0 | 0 | 0 | 1 | 5 | 4 |
|  | BQ.1.16 | 0 | 0 | 0 | 0 | 0 | 0 | 0 | 0 | 0 | 0 | 1 | 0 |
|  | BQ.1.18 | 0 | 0 | 0 | 0 | 0 | 0 | 0 | 0 | 0 | 0 | 0 | 1 |
|  | BQ.1.22 | 0 | 0 | 0 | 0 | 0 | 0 | 0 | 0 | 0 | 0 | 0 | 2 |
|  | BQ.1.23 | 0 | 0 | 0 | 0 | 0 | 0 | 0 | 0 | 0 | 0 | 0 | 1 |
|  | BQ.1.3 | 0 | 0 | 0 | 0 | 0 | 0 | 0 | 0 | 0 | 1 | 1 | 4 |
|  | BQ.1.3.1 | 0 | 0 | 0 | 0 | 0 | 0 | 0 | 0 | 0 | 0 | 1 | 0 |
|  | BQ.1.8 | 0 | 0 | 0 | 0 | 0 | 0 | 0 | 0 | 0 | 0 | 0 | 2 |

Table S4. Sub-lineages composition of Omicron BA.1 dataset.

| **Omicron BA.1** | **Total n=880 (%)** |
| --- | --- |
| BA.1.1 | 290 (32.9) |
| BA.1.17.2 | 209 (23.8) |
| BA.1/BA.1.1.529 | 160 (18.2) |
| BA.1.17 | 74 (8.4) |
| BA.1.1.1 | 37 (4.2) |
| BA.1.15 | 16 (1.8) |
| BA.1.18 | 14 (1.6) |
| BA.1.1.11 | 14 (1.6) |
| BA.1.21.1 | 12 (1.4) |
| BA.1.1.14 | 11 (1.3) |
| BA.1.20 | 9 (1.0) |
| BA.1.21 | 9 (1.0) |
| Others* | 25 (2.8) |

**sub-lineages* with prevalence lower than 1%: BA.1.10, BA.1.13, BA.1.14, BA.1.15.1, BA.1.16, BA.1.19, BA.1.1.10, BA.1.1.13, BA.1.1.15, BA.1.1.18, BA.1.1.3.

Table S5. Mutations in the BA.1 dataset. The symbol (*) indicates mutations characteristic of the variant and its descendants, and orange indicates deletions. In light blue colour are indicated mutations characteristic of specific BA.1 descendants.

| **Gene** | **Mutation** | **Total n=880 (%)** |  |  |  |  |
| --- | --- | --- | --- | --- | --- | --- |
| ORF 1a | K856R* | 830 (94.3) |  | S | T478K* | 495 (56.3) |
|  | V1887I | 285 (32.4) |  |  | E484A* | 511 (58.1) |
|  | S2083del* | 863 (98.1) |  |  | Q493R* | 492 (55.9) |
|  | L2084I* | 862 (97.9) |  |  | G496S* | 475 (54) |
|  | A2710T* | 813 (92.4) |  |  | Q498R* | 446 (50.7) |
|  | T3255I* | 850 (96.6) |  |  | N501Y* | 448 (50.9) |
|  | P3395H* | 851 (96.7) |  |  | Y505H* | 465 (52.8) |
|  | L3674del* | 849 (96.5) |  |  | T547K* | 839 (95.3) |
|  | S3675del* | 852 (96.8) |  |  | D614G* | 812 (92.3) |
|  | G3676del* | 851 (96.7) |  |  | H655Y* | 832 (94.5) |
|  | I3758V* | 811 (92.2) |  |  | N679K* | 818 (92.9) |
| ORF1b | P314L* | 879 (99.9) |  |  | P681H* | 807 (91.7) |
|  | I1566V* | 832 (94.5) |  |  | A701V | 180 (20.5) |
| S | A67V* | 843 (95.8) |  |  | N764K* | 839 (95.3) |
|  | H69del* | 843 (96.5) |  |  | D796Y* | 799 (90.8) |
|  | V70del* | 849 (96.1) |  |  | N856K* | 851 (96.7) |
|  | T95I* | 777 (88.3) |  |  | Q954H* | 851 (96.7) |
|  | G142del* | 814 (92.5) |  |  | N969K* | 812 (92.3) |
|  | VY 143-144del* | 815 (92.6) |  |  | L981F* | 809 (91.9) |
|  | Y145D* | 874 (99.3) |  | E | T9I* | 822 (93.4) |
|  | N211del | 787 (89.4) |  | M | D3G* | 757 (86) |
|  | L212I | 772 (87.7) |  |  | Q19E* | 810 (92) |
|  | G339D* | 771 (87.6) |  |  | A63T* | 848 (93.5) |
|  | R346K | 345 (39.2) |  | ORF9b | P10S* | 877 (99.7) |
|  | S371L* | 722 (82) |  |  | ENA 27-29del* | 880 (100) |
|  | S373P* | 754 (85.7) |  | N | P13L* | 880 (100) |
|  | S375F* | 727 (82.6) |  |  | ER 31-32del* | 880 (100) |
|  | K417N* | 668 (75.9) |  |  | S33del* | 872 (99.1) |
|  | N440K* | 628 (71.4) |  |  | R203K* | 845 (96) |
|  | G446S | 665 (75.6) |  |  | G204R* | 845 (96) |
|  | S477N* | 495 (56.3) |  |  |  |  |
|  |  |  |  |  |  |  |

Table S6. Sub-lineages composition of Omicron BA.2 dataset.

| **Omicron BA.2** | **Total n=1,627 (%)** |
| --- | --- |
| BA.2 | 1,124 (69.1) |
| BA.2.9 | 265 (16.3) |
| BA.2.12.1 | 66 (4.1) |
| BA.2.52 | 33 (2.0) |
| BA.2.3 | 25 (1.5) |
| BA.2.10 | 21 (1.3) |
| BA.2.22 | 18 (1.1) |
| BA.2.3.15 | 18 (1.1) |
| Others* | 57 (3.5) |

**sub-lineages* with prevalence lower than 1%: BA.2.10.1, BA.2.12, BA.2.13, BA.2.18, BA.2.23, BA.2.32, BA.2.36, BA.2.37, BA.2.44, BA.2.45, BA.2.49, BA.2.56, BA.2.58, BA.2.65, BA.2.67, BA.2.75.1, BA.2.75.2, BA.2.75.5, BA.2.3.20, BA.2.3.7, BA.2.5, BA.2.9.7.

Table S7. Mutations in the BA.2 dataset. The symbol (*) indicates mutations characteristic of the variant and its descendants, and orange indicates deletions. In light blue colour are indicated mutations characteristic of specific BA.2 descendants.

| **Gene** | **Mutation** | **Total n=1,627 (%)** |  |  |  |  |
| --- | --- | --- | --- | --- | --- | --- |
|  |  |  |  |  |  |  |
| ORF 1a | S135R* | 1,603 (98.5) |  | S | E484A* | 1506 (92.6) |
|  | T842I* | 1,465 (90) |  |  | Q493R* | 1417 (87.1) |
|  | G1307S* | 1,626 (99.9) |  |  | Q498R* | 1240 (76.2) |
|  | L3027F* | 1,618 (99.4) |  |  | N501Y* | 1277 (78.5) |
|  | T3090I* | 1,516 (93.2) |  |  | Y505H* | 1296 (79.7) |
|  | L3201F* | 1470 (90.3) |  |  | D614G* | 1512 (92.9) |
|  | T3255I* | 1621 (99.6) |  |  | H655Y* | 1618 (99.4) |
|  | P3395H* | 1624 (99.8) |  |  | N679K* | 1567 (96.3) |
|  | SG 3675-3676del* | 1627 (100) |  |  | P681H* | 1594 (98) |
|  | F3677del* | 1626 (99.9) |  |  | N764K* | 1607 (98.8) |
| ORF1b | P314L* | 1626 (99.9) |  |  | D796Y* | 1549 (95.2) |
|  | R1315C* | 1619 (99.5) |  |  | Q954H* | 1621 (99.6) |
|  | I1566V* | 1553 (95.4) |  |  | N969K* | 1566 (96.3) |
|  | T2163I* | 1534 (94.3) |  | ORF3a | H78Y | 268 (16.5) |
| S | T19I* | 1614 (99.2) |  |  | T223I* | 1618 (99.4) |
|  | LPP 24-26del | 1616 (99.3) |  | E | T9I* | 1537 (94.5) |
|  | A27S | 1614 (99.2) |  | M | Q19E* | 1562 (96) |
|  | G142D* | 1547 (95) |  |  | A63T* | 1622 (99.7) |
|  | V213G* | 1565 (96.2) |  | ORF6 | D61L* | 1523 (93.6) |
|  | G339D* | 1333 (81.9) |  | ORF9b | P10S | 1620 (99.6) |
|  | S371F* | 1506 (92.5) |  |  | EN 27-28del | 1627 (100) |
|  | S373P* | 1518 (93.3) |  |  | A29del | 1626 (99.9) |
|  | S375F* | 1491 (91.6) |  | N | P13L* | 1625 (99.9) |
|  | T376A* | 1482 (91.1) |  |  | E31del | 1626 (99.9) |
|  | D405N* | 1572 (96.6) |  |  | R32del | 1627 (100) |
|  | R408S* | 1562 (96) |  |  | S33del | 1610 (98.9) |
|  | K417N* | 1564 (96.1) |  |  | R203K* | 1625 (99.9) |
|  | N440K* | 1391 (85.5) |  |  | G204R* | 1624 (99.8) |
|  | S477N* | 1397 (85.9) |  |  | S413R* | 1549 (95.2) |
|  | T478K* | 1426 (87.6) |  |  |  |  |

Table S8. Sub-lineages composition of Omicron BA.5 dataset.

| **Omicron BA.5** | **Total n=1,761 (%)** |
| --- | --- |
| BA.5.1 | 539 (30.6) |
| BA.5.2 | 350 (19.9) |
| BA.5.2.1 | 330 (18.7) |
| BA.5.1.10 | 101 (5.7) |
| BA.5 | 49 (2.8) |
| BA.5.2.20 | 48 (2.7) |
| BA.5.1.8 | 37 (2.1) |
| BA.5.2.6 | 32 (1.8) |
| BA.5.1.5 | 29 (1.6) |
| BA.5.1.23 | 27 (1.5) |
| BA.5.2.21 | 26 (1.5) |
| BA.5.1.30 | 21 (1.2) |
| BA.5.1.22 | 17 (1.0) |
| Others* | 155 (8.8) |

**sub-lineages* with prevalence lower than 1%:BA.5.11, BA.5.1.1, BA.5.1.2, BA.5.1.3, BA.5.1.9, BA.5.1.12, BA.5.1.18, BA.5.1.21, BA.5.1.24, BA.5.1.25, BA.5.1.28, BA.5.1.33, BA.5.1.37, BA.5.2.14, BA.5.2.16, BA.5.2.19, BA.5.2.22, BA.5.2.24, BA.5.2.25, BA.5.2.26, BA.5.2.27, BA.5.2.28, BA.5.2.33, BA.5.2.34, BA.5.2.35, BA.5.2.36, BA.5.2.47, BA.5.2.53, BA.5.2.59, BA.5.2.62, BA.5.2.2, BA.5.2.3, BA.5.2.7, BA.5.2.8, BA.5.2.9, BA.5.3, BA.5.3.1, BA.5.5, BA.5.5.1, BA.5.6, BA.5.8, BA.5.9.

Table S9. Mutations in the BA.5 dataset. The symbol (*) indicates mutations characteristic of the variant and its descendants, and orange indicates deletions. In light blue colour are indicated mutations characteristic of specific BA.5 descendants.

| **Gene** | **Mutation** | **Total n=1,761 (%)** |  |  |  |  |
| --- | --- | --- | --- | --- | --- | --- |
| ORF 1a | S135R* | 1753 (99.5) |  | S | S477N* | 1589 (90.2) |
|  | T842I* | 1652 (93.8) |  |  | T478K* | 1657 (94.1) |
|  | G1307S* | 1758 (99.8) |  |  | E484A* | 1735 (98.5) |
|  | L3027F* | 1743 (99) |  |  | F486V* | 1735 (98.5) |
|  | T3090I* | 1654 (93.9) |  |  | Q498R* | 1622 (92.1) |
|  | T3255I* | 1749 (99.3) |  |  | N501Y* | 1647 (93.5) |
|  | P3395H* | 1753 (99.5) |  |  | Y505H* | 1661 (94.3) |
|  | SGF 3675-3677del* | 1760 (99.9) |  |  | D614G* | 1654 (93.9) |
| ORF1b | P314L* | 1756 (99.7) |  |  | H655Y* | 1759 (99.9) |
|  | T1050N | 367 (20.8) |  |  | N679K* | 1704 (96.7) |
|  | R1315C* | 1754 (99.6) |  |  | P681H* | 1709 (97) |
|  | I1566V* | 1732 (98.4) |  |  | N764K* | 1736 (98.6) |
|  | T2163I* | 1665 (94.5) |  |  | D796Y* | 1725 (98) |
| S | T19I* | 1753 (99.5) |  |  | Q954H* | 1758 (99.8) |
|  | L24del* | 1753 (99.5) |  |  | N969K* | 1732 (98.3) |
|  | PP 25-26del* | 1752 (99.5) |  | ORF3a | T223I* | 1747 (99.2) |
|  | A27S* | 1749 (99.3) |  | E | T9I* | 1716 (97.4) |
|  | HV 69-70del* | 1749 (99.3) |  | M | D3N* | 1732 (98.4) |
|  | F140-141del* | 1755 (99.6) |  |  | Q19E* | 1685 (95.7) |
|  | G142Y* | 1728 (98.1) |  |  | A63T* | 1756 (99.7) |
|  | V213G* | 1742 (98.9) |  | ORF9b | P10S | 1724 (97.9) |
|  | G339D* | 1495 (84.9) |  |  | D16G | 844 (47.9) |
|  | S371F* | 1622 (92.1) |  |  | EN 27-28del* | 1745 (99.1) |
|  | S373P* | 1640 (93.1) |  |  | A29del* | 1628 (92.4) |
|  | S375F* | 1603 (91.0) |  | N | P13L* | 1740 (98.8) |
|  | T376A* | 1604 (91.1) |  |  | E31del* | 1745 (99.1) |
|  | D405N* | 1724 (97.9) |  |  | R32del* | 1744 (99) |
|  | R408S* | 1686 (95.7) |  |  | S33del* | 1607 (91.2) |
|  | K417N* | 1729 (98.2) |  |  | R203K* | 1760 (99.9) |
|  | N440K* | 1590 (90.3) |  |  | G204R* | 1759 (99.9) |
|  | L452R* | 1728 (98.1) |  |  | S413R* | 1731 (98.3) |

Table S10. tMRCA estimation of the BA.1, BA.2 and B.5 isolates with the relative confidence intervals.

|  | **date** | **95%HPD^a^ lower** | **95%HPD upper** |
| --- | --- | --- | --- |
| **root** | 22/02/2020 | 13/02/2019 | 23/07/2020 |
| **BA.1** | 31/05/2021 | 15/11/2020 | 19/08/2021 |
| **BA.2** | 03/11/2021 | 01/10/2021 | 15/11/2021 |
| **BA.5** | 28/12/2021 | 29/10/2021 | 17/01/2022 |

*^a^HPD:* *highest posterior density.*


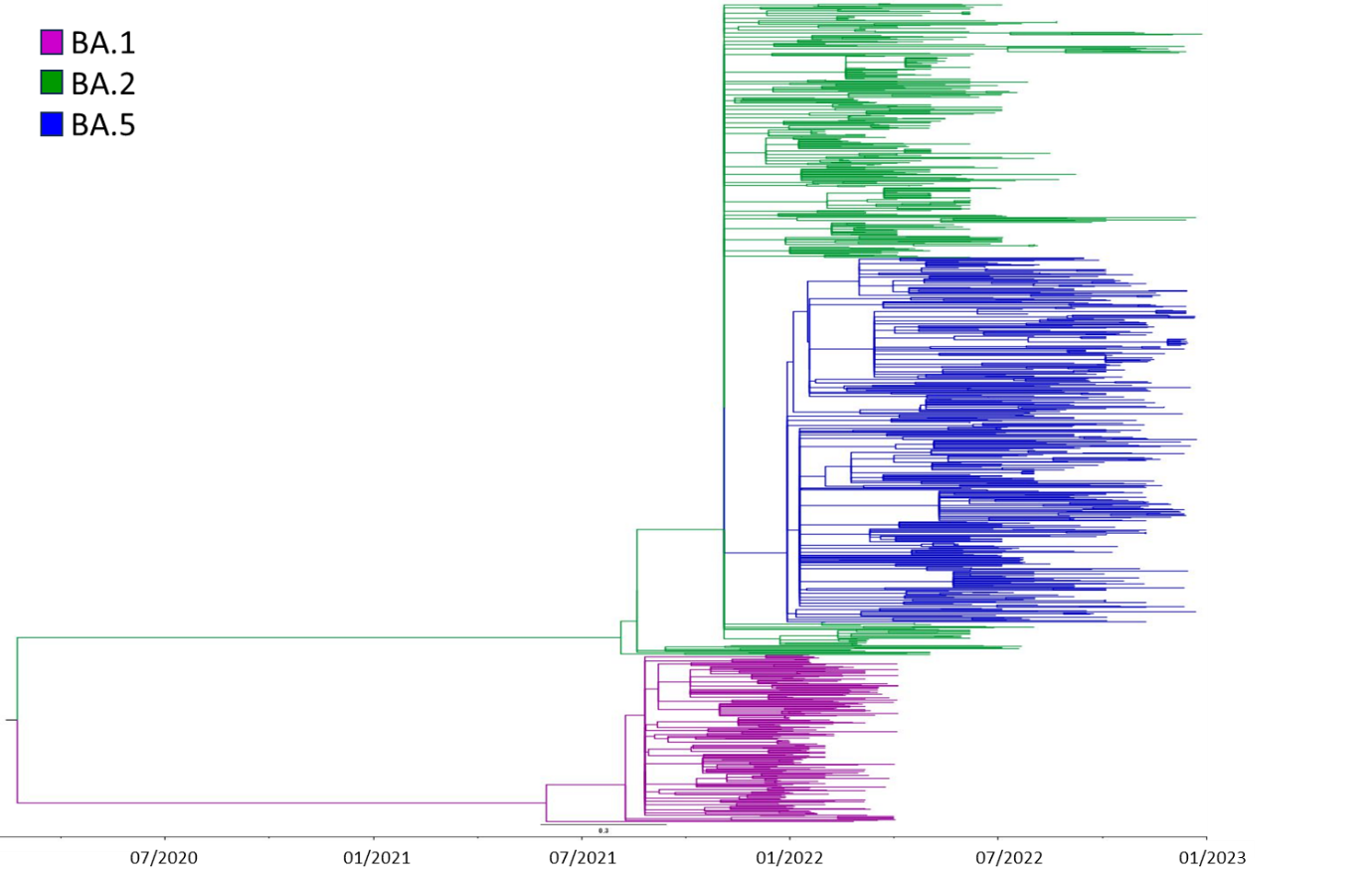


Figure S1. Maximum likelihood dated tree of BA.1, BA.2, and BA.5 Omicron variant’s clusters. The branches’ colours represent the sub-lineages (described in the legend). The time scale is described at the bottom.


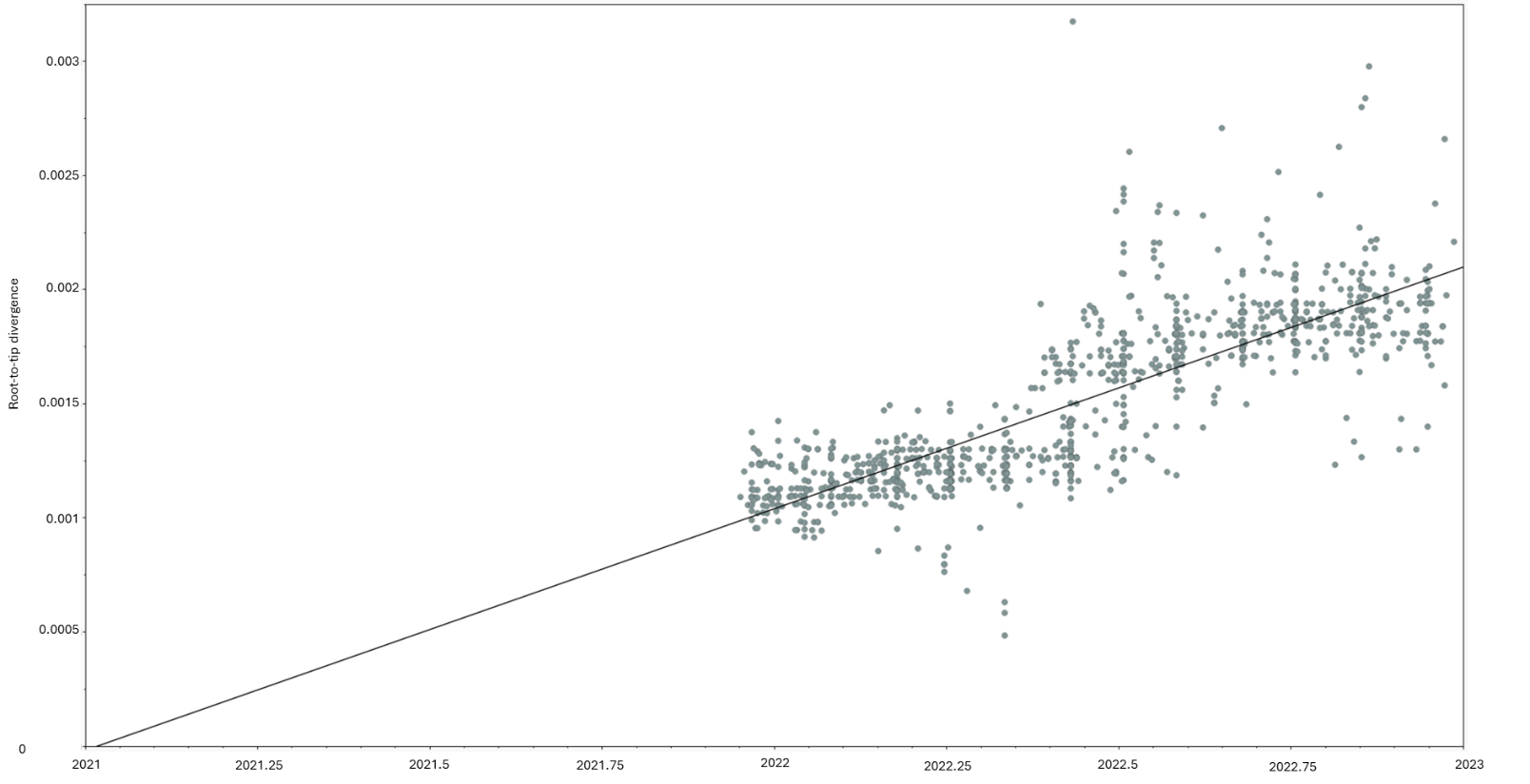


Figure S2: Root-to-tip regression analysis of Italian clusters of BA.1, BA.2 and BA.5 Omicron variants.

Figure S3: Bayesian phylogenetic tree of main BA.1 Italian clusters. The different size of the circles on the nodes indicates the posterior probability between 1 and 0.9. Different clusters are represented with different colors, numbers of clusters are reported. The scale below the tree represents the time expressed in fractions of a year.

**
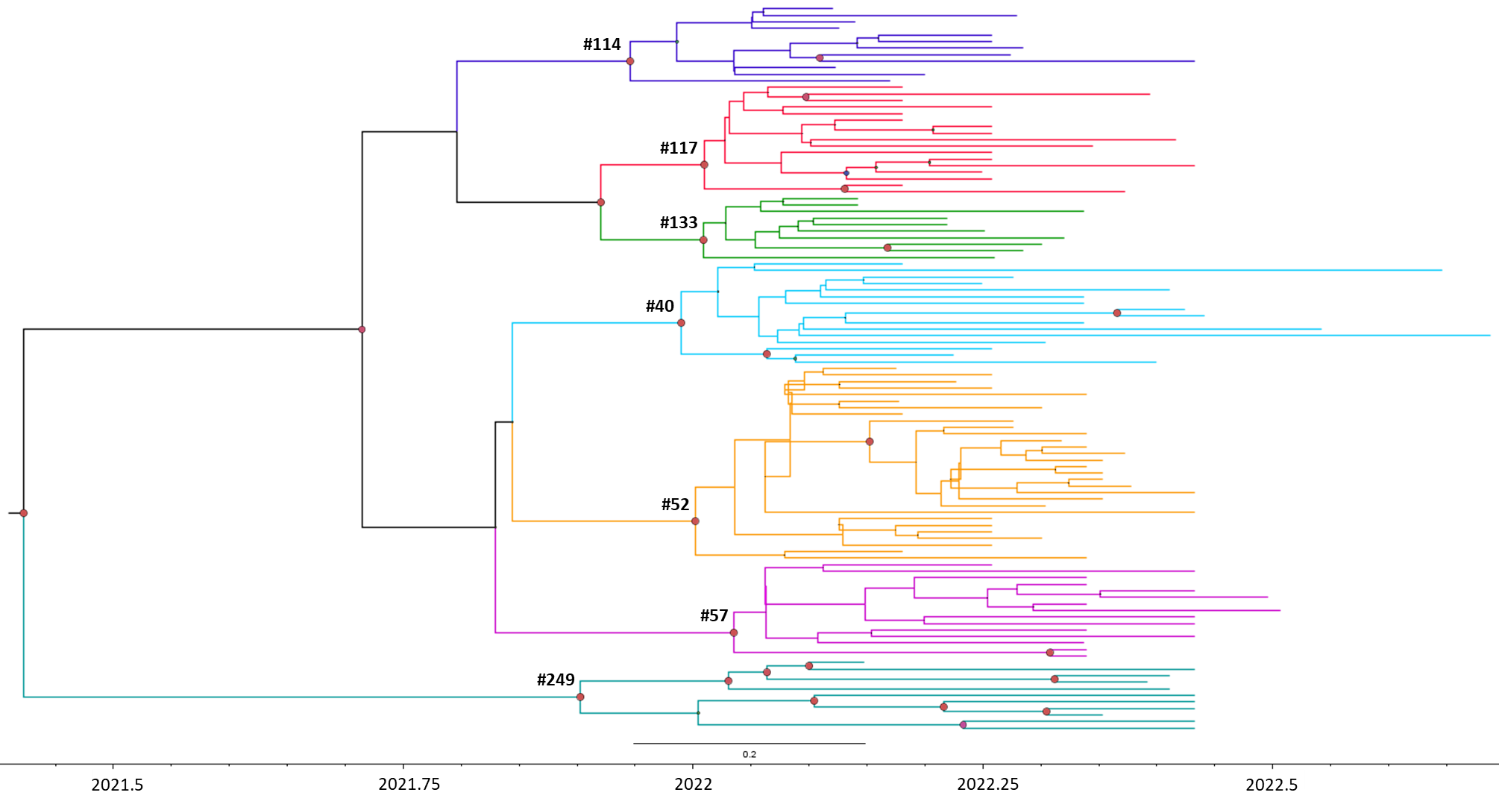
**

Figure S4: Bayesian phylogenetic tree of main BA.2 Italian clusters. The different size of the circles on the nodes indicates the posterior probability between 1 and 0.9. Different clusters are represented with different colors, numbers of clusters are reported. The scale below the tree represents the time expressed in fractions of a year.


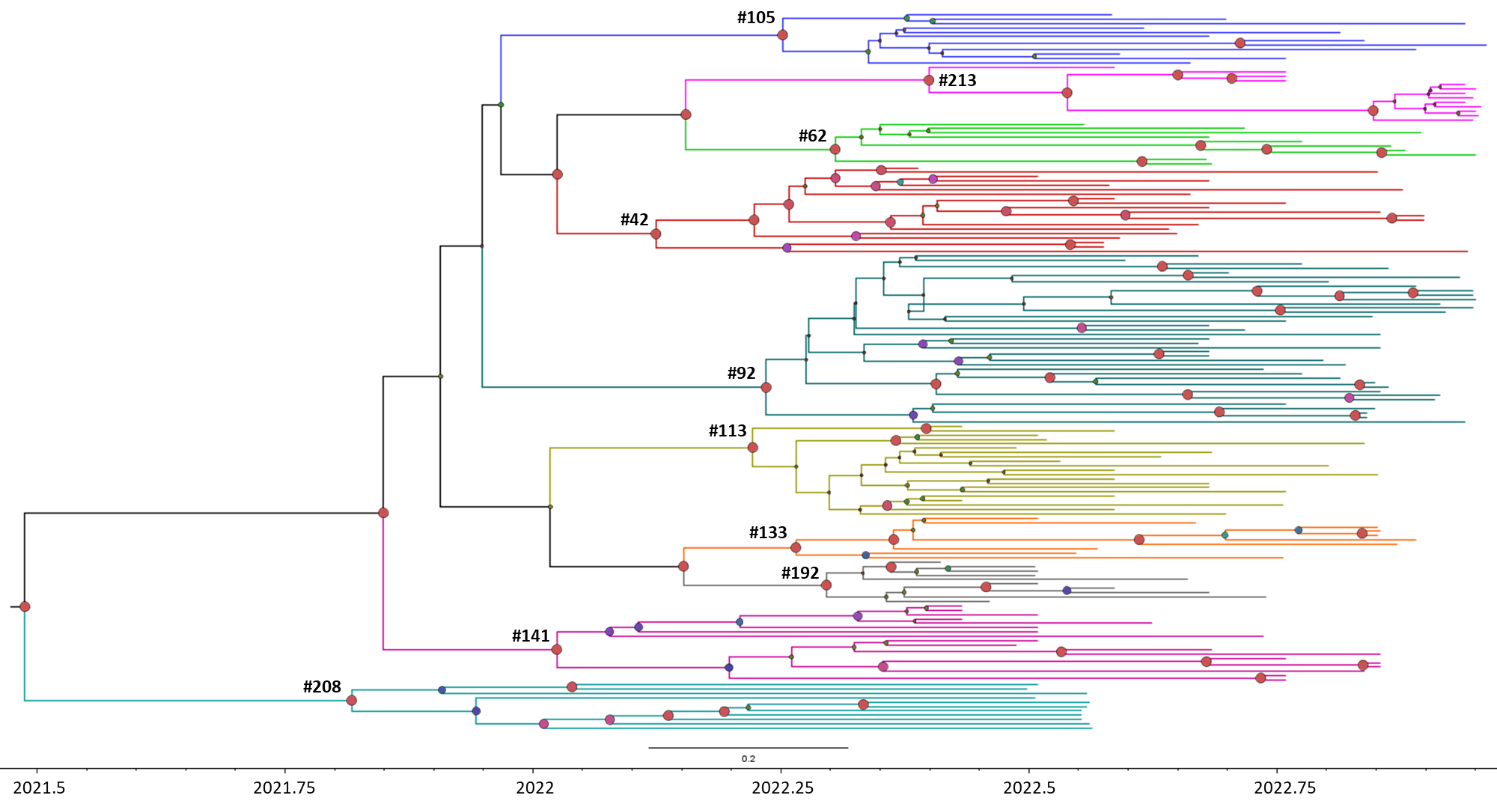


Figure S5: Bayesian phylogenetic tree of main BA.5 Italian clusters. The different size of the circles on the nodes indicates the posterior probability between 1 and 0.9. Different clusters are represented with different colors, numbers of clusters are reported. The scale below the tree represents the time expressed in fractions of a year.

**COLLABORATIVE SCIRE GROUP**

Claudia Balotta, Mario Corbellino, Massimo Galli, Valentina Ricucci, Federica Stefanelli, Nadia Randazzo, Giada Garzillo, Massimo Clementi, Maurizio Zazzi, Lia Fiaschi, Massimo Andreoni, Arianna Miola, Valeria Ricci, Laura Li Puma, Luigi Ruggerone

**Department of Biomedical and Clinical Sciences, University of Milan, Milan, Italy**

Claudia Balotta, Mario Corbellino, Massimo Galli

**Hygiene Unit, IRCCS AOU San Martino-IST, Genoa, Italy**

Valentina Ricucci, Federica Stefanelli, Nadia Randazzo

**Department of Health Sciences (DISSAL), University of Genoa, Genoa, Italy**

Giada Garzillo

**Laboratory of Microbiology and Virology, Università "Vita-Salute" San Raffaele, Milan, Italy; IRCCS San Raffaele Scientific Institute**

Massimo Clementi

**Department of Medical Biotechnologies, University of Siena, Siena, Italy**

Maurizio Zazzi, Lia Fiaschi

**Clinical Infectious Diseases, Department of System Medicine, Tor Vergata University, Rome, Italy**

Massimo Andreoni

**Intesa Sanpaolo Innovation Center- Artificial Intelligence Lab, Turin, Italy**

Arianna Miola, Valeria Ricci, Laura Li Puma, Luigi Ruggerone
